# MomL inhibits bacterial antibiotic resistance through the starvation stringent response pathway

**DOI:** 10.1002/mlf2.12016

**Published:** 2022-03-24

**Authors:** Qin Dou, Jin Yuan, Rilei Yu, Jiahui Yang, Jiayi Wang, Yuxiang Zhu, Jing Zhong, Hongan Long, Zhiqing Liu, Xianghong Wang, Yuying Li, Yichen Xiao, Jiazhen Liang, Xiao‐Hua Zhang, Yan Wang

**Affiliations:** ^1^ College of Marine Life Sciences, and Institute of Evolution & Marine Biodiversity Ocean University of China Qingdao China; ^2^ State Key Laboratory of Ophthalmology, Zhongshan Ophthalmic Center Sun Yat‐Sen University Guangzhou China; ^3^ Key Laboratory of Marine Drugs, Chinese Ministry of Education, School of Medicine and Pharmacy Ocean University of China Qingdao China; ^4^ Laboratory for Marine Ecology and Environmental Science Qingdao National Laboratory for Marine Science and Technology Qingdao China

**Keywords:** AHLs, antibiotic resistance, H2‐T6SS, *Pseudomonas aeruginosa*, starvation stringent response

## Abstract

Antibiotic resistance in gram‐negative pathogens has become one of the most serious global public health threats. The role of the *N*‐acyl homoserine lactone (AHL)‐mediated signaling pathway, which is widespread in gram‐negative bacteria, in the bacterial resistance process should be studied in depth. Here, we report a degrading enzyme of AHLs, MomL, that inhibits the antibiotic resistance of *Pseudomonas aeruginosa* through a novel mechanism. The MomL‐mediated reactivation of kanamycin is highly associated with the *relA*‐mediated starvation stringent response. The degradation of AHLs by MomL results in the inability of LasR to activate *relA*, which, in turn, stops the activation of downstream *rpoS*. Further results show that *rpoS* directly regulates the type VI secretion system H2‐T6SS. Under MomL treatment, inactivated RpoS fails to regulate H2‐T6SS; therefore, the expression of effector phospholipase A is reduced, and the adaptability of bacteria to antibiotics is weakened. MomL in combination with kanamycin is effective against a wide range of gram‐negative pathogenic bacteria. Therefore, this study reports a MomL‐antibiotic treatment strategy on antibiotic‐resistant bacteria and reveals its mechanism of action.

## Impact statement

To address the gradual ineffectiveness of new antibiotics, it is critical to highlight the effect of traditional antibiotics. Reducing or eliminating bacterial resistance by interfering with signaling pathways provides a possible solution to this problem. Our study reports a novel antibacterial strategy, MomL‐kanamycin treatment, in which MomL weakens antibiotic resistance by inhibiting the inner *N*‐acyl homoserine lactone (AHL) signaling pathway. The applicability of our treatment strategy was proven in a series of AHL‐regulated gram‐negative bacteria. Our study provides positive evidence for the feasibility of an anti‐infective strategy based on bacterial signaling pathways.

## INTRODUCTION

Bacterial resistance has become one of the most important threats to human health and has also led to the rapid failure of the existing antibiotics. It is of great value to weaken the resistance of pathogens and make them sensitive to antibiotics again. *Pseudomonas aeruginosa* (*P. aeruginosa*) is a multidrug‐resistant facultative pathogen and has been listed as a key priority bacterium by the World Health Organization^1^, and has been recognized as an opportunistic pathogen that is the most common bacterium associated with nosocomial infections, ocular surface inflammation, and ventilator‐associated pneumonia^2^. Empirical antibiotic therapy, such as tobramycin and levofloxacin^3^, improves the cure rate of severe *P. aeruginosa* infections. However, excessive use of antibiotics during treatment accelerates the development of multidrug‐resistant *P. aeruginosa* strains, leading to the ineffectiveness of empirical antibiotic therapy against this microorganism^4^. The development of new medicines against resistant bacteria is a time‐consuming process. In contrast, the combination of antibiotics and adjuvants is an economical and practical anti‐infection strategy^5^, ^6^. For instance, the combination of minocycline with other medicines could enhance its activity against the growth of *P. aeruginosa* ^7^. However, the action mechanisms of these combinations are largely unknown, and the appropriate combination to inhibit antibiotic resistance is urgently needed.

Several mechanisms for resistance development have been elucidated, including biofilm formation, efflux pump expulsion, and evolutionary mutations^6^, ^8^, ^9^, ^10^, ^11^. Signaling molecules were reported to play a key role in antibiotic resistance regulation^12^, ^13^, ^14^, ^15^, ^16^, ^17^, ^18^. Diffusible signal factors were shown to prevent *Lysobacter* spp. from being killed by increasing the antibiotic resistance of the cells^13^. Indole, as a signaling molecule, has been reported to regulate bacterial antibiotics by inducing the expression of multiple genes^13^, ^17^ *. N*‐acyl homoserine lactone (AHL) is a signaling molecule widely found in gram‐negative bacteria. AHLs are involved in various physiological behaviors, and the activity of their regulatory protein LasR is closely related to the environmental adaptability of *P. aeruginosa*, such as to salinity, heat, and heavy metal stress^19^. The LasI/R system relies on the signaling molecule 3‐*O*‐C_12_‐HSL, a member of AHLs, to increase biofilm formation and regulate the expression of alkaline protease and pyocyanin^20^. A *lasR* deletion mutation enhanced the resistance of *P. aeruginosa* to β‐lactam antibiotics^21^, ^22^. Previous studies have reported that AHLs are related to antibiotic resistance^23^. The degradation of AHLs alters the physiological behavior of bacteria^24^. The first identified AHL lactonase, AiiA, reduces the pathogenicity of *P. aeruginosa* and *Burkholderiales* by inhibiting the release of virulence factors^25^, ^26^. And MomL, a member of the metallo‐β‐lactamase family isolated from *Muricauda olearia* Th120, has been reported as an AHL lactonase in our previous studies^27^, ^28^. The degradation efficiency (*kcat*/*Km*) of MomL toward C_6_‐HSL reached 2.9 × 10^5^ s^−1^ M^−1^ and increased with chain length. This relaxed substrate selectivity confers MomL the ability to broadly inhibit the virulence of pathogens. Furthermore, compared with AiiA, MomL showed significantly higher activity, secretory ability, and stability during the treatment process of the lung cell infection^29^.

In this study, we evaluated a novel MomL‐antibiotics treatment strategy and identified the inner MomL‐inhibited AHL signaling cascade regulation pathway in *P. aeruginosa*. MomL significantly inhibits antibiotic resistance and enhances the antibacterial effect of outdated antibiotics. The effect of the MomL‐kanamycin treatment strategy was verified *in vivo* in a mouse model of *P. aeruginosa* keratitis. This study provides a new idea to restore the antibacterial activity of antibiotics.

## RESULTS

### MomL inhibits *P. aeruginosa* resistance and slows the resistance evolution by degrading AHLs

**Figure 1:**
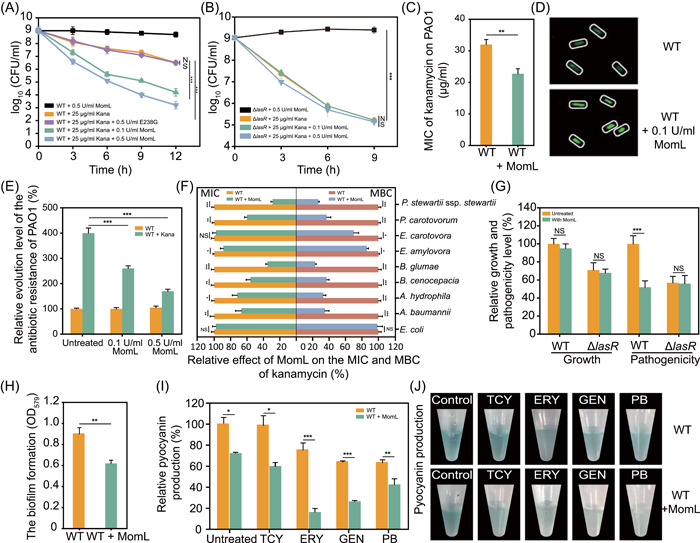
MomL inhibits *Pseudomonas aeruginosa* PAO1 resistance and decreases virulence factor production and biofilm formation. (A) MomL reduced the antibiotic resistance of *P. aeruginosa* PAO1 to kanamycin. Compared with that in the kanamycin alone group and the E238G‐kanamycin group, the antibiotic resistance of PAO1 was significantly inhibited in both two MomL‐treated groups. The effect in the 0.5 U/ml MomL treatment group was better. (B) In Δ*lasR*, the antibiotic resistance to kanamycin was unaffected with MomL. (C) MIC of kanamycin in the presence or absence of MomL. (D) Fluorescence imaging assay of kanamycin transport by the wild‐type strain with or without 0.1 U/ml MomL. (E) Evolution of the antibiotic resistance of *P. aeruginosa* PAO1 induced by different MomL‐kanamycin combinations or the corresponding single components through a continuous repeated transfer assay. (F) The effect of MomL on the MIC and MBC of kanamycin against the selected gram‐negative pathogens, namely *Pantoea stewartii* subsp. *stewartii* DSM30176, *Pectobacterium carotovorum* BNCC138474, *Erwinia carotovora* BNCC166445, *Erwinia amylovora* ATCC51855, *Burkholderia glumae* BNCC341645, *Burkholderia cenocepacia* BNCC157156, *Aeromonas hydrophila* YC57, and *Acinetobacter baumannii* YC28. *Escherichia coli* was used as a negative control. (G) The growth and pathogenicity detection of *P. aeruginosa* PAO1 and Δ*lasR* after 24 h of cultivation in the absence of MomL or presence of 0.1 U/ml MomL. The pathogenicity was measured by the survival rate of zebrafish. (H) *P. aeruginosa* PAO1 biofilm detection without or with MomL treatment. (I, J) Pyocyanin production by *P. aeruginosa* PAO1 under 1/2 MIC antibiotic (in the presence or absence of MomL), namely tetracycline (TCY), erythromycin (ERY), gentamicin (GEN), or polymyxin B sulfate (PB). Error bars show the standard deviation of three replicates. NS, not significant; *p < 0.05; **p < 0.01; ***p < 0.001. All data are mean ± SEM. MIC, minimum inhibitory concentration.

*P. aeruginosa*, an ESKAPE pathogen^37^, ^38^, shows variable levels of resistance to a range of traditional antibiotics, including kanamycin used for the following assays. MomL, an AHL lactonase, showed the activity to enhance bacterial sensitivity to kanamycin (Figure 1A). The survival rate of *P. aeruginosa* PAO1 was 10% over 6 h after treatment with 25 μg/ml kanamycin. After 12 h under the same conditions, the rate was approximately 0.1%. In contrast, the survival rate was only approximately 0.1% after 6 h and 0.001% after 12 h under 0.1 U/ml MomL, and the rate further decreased to approximately 0.0001% after 12 h under 0.5 U/ml MomL. *P. aeruginosa* PAO1 under MomL treatment showed considerably increased sensitivity to kanamycin, suggesting that MomL could inhibit the antibiotic resistance of *P. aeruginosa* PAO1 (Figure 1A). The inefficacy of E238G (the inactive MomL mutant protein^39^ that could not degrade AHLs) on antibiotic resistance verified that MomL inhibited bacterial resistance to kanamycin by degrading AHLs (Figure 1A). For Δ*lasR*, whose AHL pathway was blocked, the presence or absence of MomL did not affect the sensitivity of cells to kanamycin (Figure 1B). Considering the significant inhibition of antibiotic resistance under 0.1 U/ml MomL treatment, we chose 0.1 U/ml as the main experimental concentration in subsequent *in vitro* studies. All these results showed that MomL could significantly improve the efficacy of kanamycin by efficiently degrading AHLs (Figure S1). Our further minimum inhibitory concentration (MIC) analyses showed that the kanamycin resistance of *P. aeruginosa* PAO1 under MomL treatment was reduced to 70% of that under kanamycin treatment alone (Figure 1C). To verify the universality of this inhibition, MIC assays of other antibiotics on *P. aeruginosa* PAO1 were performed. Their MICs were reduced by 2–4 times under MomL‐treated conditions (Table S1). Furthermore, the concentration of antibiotics entering the cells was monitored with fluorescently labeled kanamycin, and the results indicated that the presence of MomL significantly enhanced antibiotic entry into the cells (Figures 1D and S2). To explore whether MomL could inhibit the evolution of antibiotic resistance, we carried out continuous repeated transfer experiments with *P. aeruginosa* PAO1 in the presence of the kanamycin‐MomL combination or the corresponding single chemical. To maximize the level of antibiotic resistance in the evolving populations, we gradually increased the kanamycin concentration during the repeated transfer process. After 90 generations, the MIC levels of *P. aeruginosa* PAO1 increased four‐folds, compared with those of its ancestor in the presence of kanamycin alone. In contrast, the MomL‐kanamycin combination significantly slowed the evolution of antibiotic resistance. The inhibition of antibiotic resistance evolution was found to be MomL dose‐dependent (Figure 1E). In addition to *P. aeruginosa* PAO1, we also detected eight AHL‐containing, gram‐negative, pathogenic bacteria, including the ESKAPE pathogen *Acinetobacter baumannii* and widespread pathogens in natural and clinical environments. [Correction added on November 5, 2022, after first online publication: In the previous sentence, “nine AHL‐containing, gram‐negative, pathogenic bacteria, …” was changed to “eight AHL‐containing, gram‐negative, pathogenic bacteria, …”.] Except the MIC of *Erwinia carotovora* (*E. carotovora*), all other bacteria showed markedly increased sensitivity to conventional antibiotics when they were combined with MomL. In contrast, MomL could not inhibit antibiotic resistance in bacteria that do not contain AHL signaling pathways, such as *E. coli* (Figure 1F).

Moreover, MomL could reduce the pathogenicity of *P. aeruginosa* PAO1 to about 50% (minimum reached 39%) and decrease the biofilm level to about 65% (Figure 1G,H). [Correction added on November 5, 2022, after first online publication: In the previous sentence, “39%” was changed to “about 50% (minimum reached 39%)” and “65%” was changed to “about 65%”.] Bacterial biofilms can reduce the penetration of antibiotics by forming a physical barrier. The inhibition of bacterial biofilm formation by MomL made antibiotics more accessible to bacterial cells, which was another potential pathway of antibiotic resistance inhibition by MomL. In addition, under MomL treatment, the pathogenicity of *P. aeruginosa* PAO1 to zebrafish was reduced, while bacterial growth was unaffected. These effects also disappeared in Δ*lasR* (Figure 1G). Specifically, MomL significantly reduced the production of pyocyanin, a major virulence factor of *P. aeruginosa*, regardless of the presence or absence of antibiotics (Figure 1I,J). Therefore, all these results indicated that with the function of degrading AHLs, MomL could inhibit antibiotic resistance, and the MomL‐kanamycin combination could be developed as a novel anti‐infection strategy that minimized the generation of resistant bacteria.

### *relA*‐mediated starvation stringent responses are involved in the MomL‐induced inhibition of antibiotic resistance

**Figure 2:**
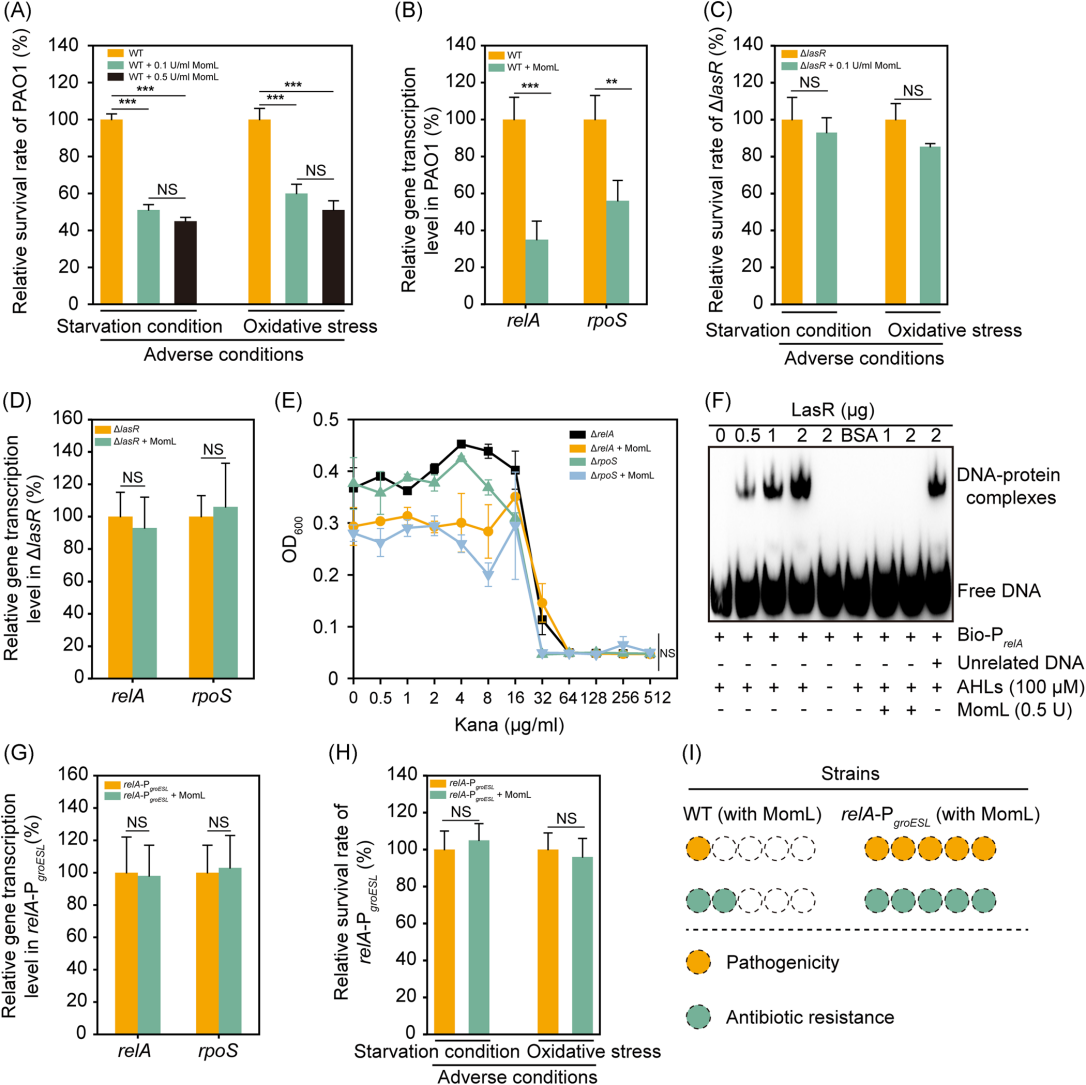
*relA*‐mediated starvation stringent responses are involved in the inhibition of antibiotic resistance development. (A) Survival rate of *Pseudomonas aeruginosa* PAO1 with different concentrations of MomL under starvation stress and oxidative stress. It was significantly inhibited under MomL treatment. But the effect of the 0.1 U/ml MomL treatment group was similar to that of the 0.5 U/ml MomL treatment group. (B) Real‐time PCR assays of the relative expression levels of *relA* and *rpoS* in *P. aeruginosa* PAO1. They were downregulated under MomL treatment. (C) Survival rate of Δ*lasR* under starvation stress and oxidative stress. (D) Real‐time PCR assays of the relative expression levels of *relA* and *rpoS* in Δ*lasR*. The expression level of them was unaffected with MomL. (E) The MIC assay to kanamycin of Δ*relA* and Δ*rpoS*. For Δ*relA* and Δ*rpoS*, their antibiotic resistances were unaffected with MomL. (F) EMSA was performed with serial dilutions of LasR ranging from 0 to 2 μg with the promoter of *relA*. MomL was added to the reaction system to detect the effect on the combination of LasR and the *relA* promoter. The signaling molecule AHL was necessary for this combination. As the control, BSA verified the specificity of this interaction. (G) Relative expression levels of *relA* and *rpoS* in the *relA*‐P_*groEL*_ mutant in the absence or presence of MomL. (H) Survival rate of the *relA*‐P_*groEL*_ mutant in the absence or presence of MomL under starvation stress and oxidative stress. The connection between MomL and these responses disappeared. (I) Schematic representation of the ability of MomL treatment to inhibit the antibiotic resistance and pathogenicity of the wild type and *relA*‐P_*groE*_. Error bars show the standard deviation of three replicates. NS, not significant; **p​​​​​​ < 0.01; ***p < 0.001. All data are mean ± SEM. BSA, bovine serum albumin; EMSA, electrophoretic mobility shift assay.

Previous studies have reported that the adversity responses, such as the nutrient starvation response, might enhance antibiotic resistance^40^, ^41^. To reveal the inner mechanism of MomL‐induced inhibition of antibiotic resistance, a series of adversity survivability tests under different conditions were performed. Under the starvation condition and the oxidative stress, the effect of MomL was most significant. The survival rates of *P. aeruginosa* PAO1 under these two conditions were significantly reduced by MomL (Figure 2A). Considering that the *relA* gene encodes a (p)ppGpp synthetase in the starvation stringent response pathway and that the *rpoS* gene (regulated by *relA*) is a key gene in the oxidative stress response pathway^42^, a quantitative PCR assay on *relA* and *rpoS* was performed. The result showed that the transcription levels of these two genes were downregulated by MomL (Figure 2B). The differential expression of these two genes was in accordance with the above survival results. To confirm the role of MomL‐mediated degradation of AHLs in this regulation, tests on Δ*lasR* were performed. For Δ*lasR*, the survival ability under the above two conditions and the transcription level of these two genes were unaffected with MomL (Figure 2C,D). The inhibition of antibiotic resistance by MomL disappeared in Δ*relA* and Δ*rpoS* (Figure 2E). In the subsequent EMSA, the *in vitro* binding between LasR and the promoter of *relA* was observed (Figure 2F). This binding depended on the presence of AHLs. The presence of MomL obviously stopped this binding. The BSA control and the unrelated DNA control showed binding specificity. In further studies, the *relA* promoter was replaced with P_*groESL*_, a constitutive high‐expression promoter, preforming the strain *relA*‐P_*groESL*_. The reason why we chose this promoter was that the P_*groESL*_‐regulated gene *groEL* was unaffected with the addition of MomL (Figure S3). In *relA*‐P_*groESL*_, LasR could not regulate the expression of *relA*. The transcription levels of *relA* and *rpoS* were unaffected with MomL treatment (Figure 2G). In addition, the survival rates of MomL‐treated *relA*‐P_*groESL*_ under the starvation condition and the oxidative stress were the same as those of the untreated group (Figure 2H). This result was consistent with the expression levels of *relA* and *rpoS* (Figure 2G). Due to the lack of interaction between LasR and the *relA* promoter in *relA*‐P_*groESL*_, the inhibition of pathogenicity and antibiotic resistance by MomL disappeared (Figure 2I). Therefore, our results indicated that *relA*‐mediated starvation stringent responses are involved in the MomL inhibition of antibiotic resistance.

### H2‐T6SS is directly regulated by RpoS and participates in MomL‐mediated inhibition of antibiotic resistance

To further explore the biological mechanism underlying the downstream pathway of antibiotic resistance inhibition by MomL, genome‐wide transcriptional profiling was performed. A series of efflux pump genes were regulated by MomL (Figure S4), including *opmF*, encoding an outer membrane efflux family protein; *tetR*, encoding a tetracycline resistance repressor protein; and *agtA*, encoding a polyamine ABC transporter ATP‐binding protein. In addition, the biosynthetic gene cluster of phenazine, the precursor substance of pyocyanin, was downregulated more than 100 folds (Figure S5A,B), which confirmed the results described in Figure 1I,J.

**Figure 3:**
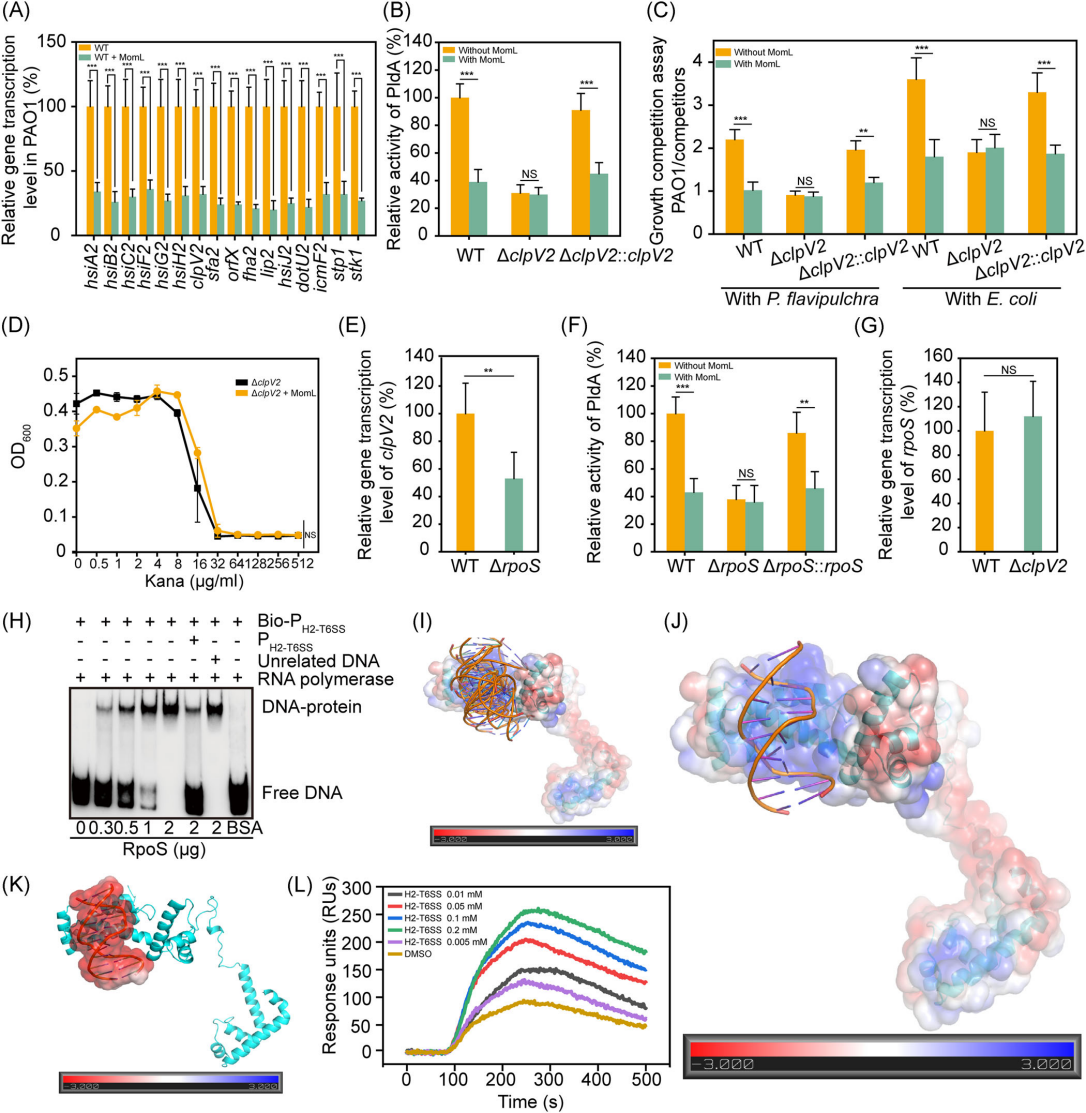
H2‐T6SS is involved in MomL‐mediated inhibition of resistance development. (A) Real‐time PCR assays of the relative expression levels of the H2‐T6SS gene cluster in *Pseudomonas aeruginosa* PAO1. As the control, the expression of *groEL* was unaffected with MomL (Figure S3). (B) PldA activity of the wild‐type strain, Δ*clpV2* and Δ*clpV2*::*clpV2* in the presence or absence of MomL. (C) Growth competition assay of *P. aeruginosa* PAO1 with competitors. The wild‐type strain, Δ*clpV2* and Δ*clpV2*::*clpV2* cocultured with *P. flavipulchra* NCIMB 2033^T^ (left) and *Escherichia coli* DH5*α* carrying pUCm‐T (right) in the presence or absence of MomL. (D) The MIC assay of kanamycin against Δ*clpV2*. For Δ*clpV2*, its antibiotic resistance was unaffected with MomL. (E) Real‐time PCR assays of the relative expression levels of *clpV2* in the wild‐type strain and Δ*rpoS*. (F) PldA activity in the wild‐type strain, Δ*rpoS* and Δ*rpoS*::*rpoS*. (G) Real‐time PCR assays of the relative expression levels of *rpoS* in the wild‐type strain and Δ*clpV2*. (H) Binding of RpoS and the promoter of H2‐T6SS. As the control, BSA verified the specificity of this interaction. (I) Computationally determined binding modes of DNA at RpoS with the top 10 ranking scores. (J,K) The highest docking conformation of DNA at the binding site of RpoS, with the surface electrostatic potentials of RpoS and DNA shown. (L) The time response curve for the binding of DNA with RpoS from surface plasmon resonance (SPR) testing. Error bars show the standard deviation of three replicates. NS, not significant; *p < 0.05; **p < 0.01; ***p < 0.001. All data are mean ± SEM.

Notably, a cluster of 16 genes annotated as the type VI secretion system H2‐T6SS was significantly downregulated by MomL (Figures 3A and S6A,B). The presence of MomL significantly reduced the expression of PldA, the effector of H2‐T6SS, and the activity of PldA was reduced to 40% of that in the wild type. Knocking out the key gene *clpV2* of H2‐T6SS, encoding the ATPase of the type VI secretion system, significantly reduced the activity of PldA regardless of the presence of MomL, while the *clpV2* complementarity restored the activity of PldA (Figure 3B). As PldA is one of the virulence factors of *P. aeruginosa* PAO1, a survival competition experiment was carried out. To visually determine the survival rate of different strains, two strains were selected, namely *Pseudoalteromonas flavipulchra* (*P. flavipulchra*) NCIMB 2033^T^ and the engineered strain *E. coli* DH5*α* carrying the pUCm‐T plasmid. The results showed that MomL significantly reduced the survival rate of *P. aeruginosa* PAO1. After the *clpV2* gene was knocked out, the competitiveness of *P. aeruginosa* PAO1 was reduced regardless of the presence of MomL. If *clpV2* was complemented, competitiveness was restored (Figure 3C). The results showed that MomL reduced the survival competitiveness of *P. aeruginosa* PAO1 by inhibiting H2‐T6SS. MomL lost the ability to inhibit antibiotic resistance in the mutant Δ*clpV2* (Figure 3D). Moreover, the expression of *clpV2* in the Δ*rpoS* was reduced to 50% of that in the wild type (Figure 3E). Consistently, the activity of PldA was also reduced to 40% in Δ*rpoS*, which was restored in the *rpoS* complementary mutant (Figure 3F). However, the expression of the *rpoS* gene was not affected by *clpV2* knockout (Figure 3G). The above results show that RpoS regulates *clpV2*. To explore its regulation mode, the EMSA was performed. The result showed that RpoS is directly bound to the 100 bp promoter sequence of H2‐T6SS (Figure 3H). In addition, we docked the promoter of H2‐T6SS to the RpoS model using global blind docking in HDOCK. The top 10 conformations of the DNA fragment were well clustered together at the cleft of the N‐terminal domain of RpoS, where high positive electrostatic potential was observed (Figure 3I). The promoter of H2‐T6SS possesses a negative electrostatic potential (Figure 3J,K), which is complementary to the positive electrostatic potential at the binding cleft of RpoS. Thus, the electrostatic interaction is predicted to be the main driving force for the interactions between the DNA and RpoS. Then, the binding affinity of the H2‐T6SS promoter to RpoS was tested based on SPR. The result from SPR testing suggested that the DNA could strongly bind with RpoS with an equilibrium association constant (*K*_D_) of 87.6 nM (Figure 3L). Overall, both computational docking and experimental testing studies suggest that the promoter of H2‐T6SS can favorably interact with RpoS with high binding affinity. Therefore, the H2‐T6SS gene cluster was directly regulated by RpoS, and the presence of MomL led to decreased expression of the effector PldA, which caused changes in the stress adaptation behavior of *P. aeruginosa* PAO1, such as a reduction in antibiotic resistance. Further results showed that H1‐T6SS and H3‐T6SS were not involved in this regulatory pathway (Figure S7).

### MomL enhances the efficacy of poorly effective antibiotics on bacterial keratitis *in vivo*

**Figure 4:**
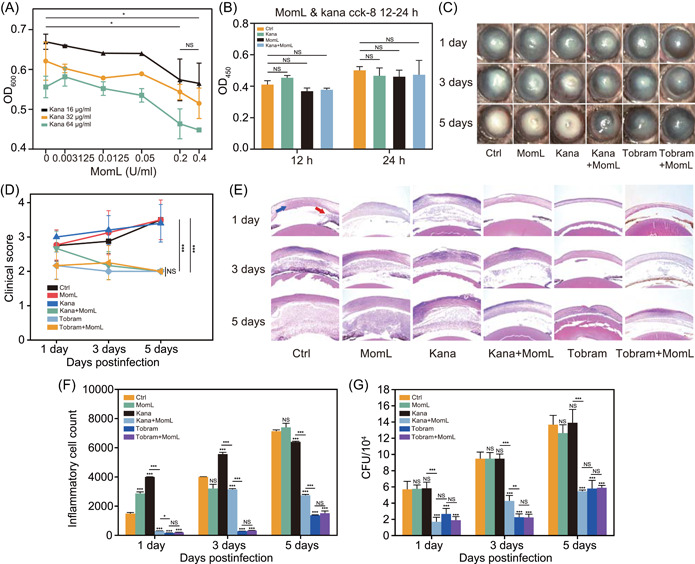
MomL inhibits *Pseudomonas aeruginosa* resistance to increase the efficacy of kanamycin *in vivo* and *in vitro*. (A) The MIC test of MomL with different concentrations of kanamycin (16, 32, and 64 μg/mL) against *P. aeruginosa*. As the control, MomL alone has almost no bacterial inhibitory ability (Figure S8A). (B) With the cell counting kit‐8 assay, HCECs were treated with MomL, kanamycin, and MomL‐kanamycin for 12 and 24 h to assay their cell viability. Tobramycin, a commonly used clinical antibiotic for the treatment of keratitis, was used as a positive control (Figure S8B). (C,D) Representative ocular pictures and clinical scores in the control, kanamycin, MomL, MomL‐kanamycin, tobramycin, and MomL‐tobramycin (Figure S8C) treatment in the *P. aeruginosa* PAO1 animal model at 1, 3, and 5 days. Magnification: ×16. (E) The histopathology of the control, kanamycin, MomL, MomL‐kanamycin, tobramycin, and MomL‐tobramycin groups at 1, 3, and 5 days. Magnification: ×100. The blue arrow indicates inflammatory cells infiltrating the corneal tissue. [Correction added on November 5, 2022, after first online publication: In the previous sentence, “Magnification: ×40” was changed to “Magnification: ×100”.] The red arrow indicates inflammatory cells invading the anterior chamber. (F) The inflammatory cell count of the whole cornea in the HE staining of the control, kanamycin, MomL, MomL‐kanamycin, tobramycin, and MomL‐tobramycin treatments at 1, 3, and 5 days. (G) Viable bacterial loads in colony‐forming units (CFUs) of the control, kanamycin, MomL, MomL‐kanamycin, tobramycin, and MomL‐tobramycin groups at 1, 3, and 5 days. Error bars show the standard deviation of three replicates. NS, not significant; *p < 0.05; **p < 0.01; ***p < 0.001. All data are mean ± SEM. HCEC, human corneal epithelial cells; HE, hematoxylin and eosin.

To further determine the inhibition of *P. aeruginosa* infection *in vivo* by the combination of MomL and kanamycin, we determined the effective concentration of MomL‐kanamycin and its biosafety. The concentration of MomL above 0.05 U/ml induced its positive inhibitory effect on antibiotic resistance *in vitro* (Figure 4A). Treatment of HCECs with kanamycin (640 μg/ml), MomL (2 U/ml), and their combination for 12 and 24 h showed no difference in compatibility between treated and untreated cells; thus, MomL‐kanamycin had good biosafety at therapeutic concentrations (Figure 4B). Based on the above results, we chose 2 U/ml as the main experimental concentration of MomL in the following *in vivo* assays.

To evaluate the efficacy of MomL *in vivo*, we established a mouse corneal model of *P. aeruginosa* infection. Mice were treated with kanamycin, MomL, and their mixture, and the eyes of mice were observed under a clinical slit lamp on days 1, 3, and 5 and evaluated pathological features (Figure 4C). Based on the extent and depth of corneal opacity, the clinical scores on mouse eyes were obtained (Figure 4D). Kanamycin alone and MomL alone showed no therapeutic effect, while the treatment effect of MomL‐kanamycin was significant and was close to the effect of the tobramycin control (the commonly used clinical antibiotic for the treatment of bacterial keratitis^43^) (Figure 4D). The results of HE staining (Figure 4E) of the corneal slices were consistent with the clinical scores. Compared with the results of the untreated control, kanamycin alone control and MomL alone control, MomL‐kanamycin and tobramycin (alone or with MomL) significantly reduced symptoms, including corneal edema, epithelium defects, and stromal inflammatory cell infiltration (Figure 4E). Based on these results, inflammatory cell counting was performed (Figure 4F). The result showed that MomL‐kanamycin and tobramycin (alone or with MomL) significantly inhibited the formation of inflammatory cells (Figure 4F). Furthermore, the result of the bacterial loading test showed that MomL‐kanamycin effectively inhibited bacterial proliferation *in vivo* (Figure 4G), and its effect was nearly equal to the effect of tobramycin (alone or with MomL). Therefore, MomL could restore the antibacterial activity of kanamycin in the treatment of bacterial keratitis, and its effect was equal to that of tobramycin, the currently used clinical drug. In addition, to further verify the effect of MomL on other antibiotics that had poor performance, the same tests on cefazolin and neomycin were performed^44^, ^45^, ^46^, ^47^. For these two antibiotics, their effects on the inhibition of antibiotic resistance *in vitro*, pathological features, clinical scores, related symptoms, and inflammatory cells were significantly enhanced by MomL (Figures S9 and 10).

## DISCUSSION

Bacterial resistance results in the failure of anti‐infective treatment and additional economic costs. At the same time, we are facing a growing shortage of effective antibiotics, especially for the treatment of gram‐negative‐resistant bacteria. Therefore, it is of great significance to develop novel strategies for the treatment of resistant bacterial infections. Here, we report a novel antibacterial strategy, MomL‐antibiotic treatment, which delays the development of bacterial antibiotic resistance. This strategy is based on the MomL‐mediated inhibition of AHL signaling regulation. The effects of AHL signaling molecules, as one of the earliest discovered signaling molecules, on a variety of physiological behaviors of bacteria have been widely studied. However, previous studies have focused mainly on the relationship between AHLs and the pathogenicity, virulence factor production, and environmental adaptability of bacteria^20^, ^48^, ^49^, ^50^, ^51^, ^52^. The relationship between the AHL signaling pathway and antibiotic resistance is largely unknown. Our results provide a new mechanism regarding the use of AHL lactonase as an adjuvant in combination with approved antibiotics for the treatment of infections caused by antibiotic‐resistant bacteria.

**Figure 5:**
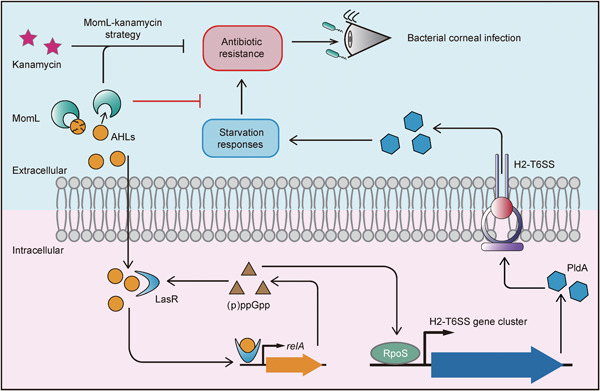
The MomL‐mediated inhibition mechanism of bacterial resistance. AHL, *N*‐acyl homoserine lactone.

In this study, we found that MomL‐induced antibiotic resistance inhibition was coupled with *relA*‐induced starvation responses (Figure 5). Previous studies reported correlations between various bacterial stress responses and antibiotic resistance^6^, ^53^, ^54^, ^55^, ^56^. However, the mechanisms of *relA*‐mediated stress responses to antibiotic resistance are poorly understood. The *relA* gene can positively regulate LasR expression through (p)ppGpp^57^. At the same time, the key gene *rpoS* in the oxidative stress pathway is also regulated by the *relA*‐mediated starvation stress pathway^53^, ^58^. Our results supported these conclusions, and we found that LasR could in turn positively regulate *relA* and further enhance the expression of *rpoS*. We also found that MomL inactivated LasR by degrading AHLs, thereby inhibiting the *relA‐rpoS* pathway and blocking the signal transmission towards H2‐T6SS. [Correction added on November 5, 2022, after first online publication: In the previous sentence, “transmitting signals to” was changed to “blocking the signal transmission towards”.] It was reported that H2‐T6SS is regulated by AHL‐mediated quorum sensing and iron deficiency^59^, and RpoS regulated the expression of T6SS in *Yersinia pseudotuberculosis* ^60^. It was speculated that LasR might regulate H2‐T6SS^59^. Our results were consistent with this speculation and revealed that H2‐T6SS was located downstream of *relA* and *rpoS* and was directly regulated by RpoS. The detailed mechanism by which RpoS regulates the H2‐T6SS needs further investigation. Moreover, our further evidence showed that the pathway we revealed also existed in the actual infection process (Figure S11).

We tested the efficacy of this novel strategy in the treatment of bacterial keratitis. There are two reasons for performing this assay. One reason is that only a few studies have focused on the efficacies of AHL‐degrading enzymes in the disease models clinically. The second reason is the need for new strategies to treat keratitis caused by drug‐resistant bacteria. Corneal infection caused by *P. aeruginosa* develops rapidly, triggering an inflammatory response that may lead to vision loss and poor prognosis clinically^61^. Fluoroquinolone antibiotics, such as levofloxacin, gatifloxacin, and moxifloxacin, and aminoglycoside antibiotics, such as tobramycin and levofloxacin, are the most effective antibiotics for *P. aeruginosa* therapy^62^. However, increasing numbers of drug‐resistant isolates have been reported over the past two decades^63^. In our study, we reported that MomL had good biocompatibility at the therapeutic concentration and showed an ideal adjuvant therapeutic effect on antibiotics to combat bacterial keratitis caused by *P. aeruginosa*. The MomL‐antibiotic strategy has been verified as an effective treatment for bacterial infection by restoring the antibacterial ability of poorly effective antibiotics, such as kanamycin, cefazolin, and neomycin. However, the effect of MomL on tobramycin was not significant. The reason could be that the antibacterial effect of tobramycin was significant enough to make the effect of MomL be ignored. Furthermore, MomL exerted strong auxiliary antibacterial activity and low toxicity to HCECs, indicating that it might be suitable for *P. aeruginosa* infection clinically^64^.

It takes considerable resources and time to develop new antibiotics, which will gradually become ineffective during usage. Reducing or eliminating bacterial resistance by interfering with signaling pathways can make traditional antibiotics effective again and largely prevent the emergence of more complex resistance. In future studies, it is worth deeply studying this regulatory pathway to identify new key gene targets and provide a theoretical basis for the development of new adjuvants. In addition, although clinical trials have been conducted and the efficacy of MomL in the treatment of infection has been demonstrated, the limitations of the clinical application of protein drugs remain, including easy degradation by proteases and unstable activity^65^, ^66^. Further studies will be conducted to improve the efficacy and dosage form of MomL, such as controlling MomL release through drug delivery systems, and additional clinical trials will be conducted to confirm its safety, efficacy, and clinical applicability. Moreover, additional applications of MomL could be developed, such as using synthetic biotechnology to express MomL in antibiotic‐producing bacteria or intestinal probiotic yeast. This application can not only solve the unstable feature of MomL to some extent but can also eventually lead to a clinical treatment or prevent bacterial infections.

In summary, by degrading the bacterial signaling molecule AHLs, MomL inhibits the activity of LasR (Figure 5). The interaction between AHLs and LasR activates the expression of *relA*. Thus, *relA* expression is reduced, and downstream RpoS activity is inhibited. For the function of RpoS to turn on the expression of the H2‐T6SS gene cluster, PldA production and the subsequent starvation stress and oxidative stress responses were inhibited. Finally, the formation of antibiotic resistance based on these responses is decreased. This MomL‐kanamycin treatment strategy has a significantly positive effect on the alleviation of bacterial corneal infection and is expected to be applied in clinical treatment.

## MATERIALS AND METHODS

### Bacterial strains, plasmids, and general methods

*P. aeruginosa* PAO1 and the derived mutants were grown in a 40% TSB medium. The concentration of MomL in the experiments was 0.1 or 0.5 U/ml. *Escherichia coli* strain DH5*α* was used for DNA manipulation. Additional bacterial strains and plasmids used in this study are described in Table S2. DNA fragmentation and plasmid extraction were carried out following the instructions of kits purchased from Omega (Plasmid Mini Kit I and Gel Extraction Kit). Polymerase chain reaction (PCR) primers were synthesized by Tsingke Biological Technology Company. Molecular manipulations were performed according to methods described previously^14^, ^30^, ^31^. Other molecular biology reagents and restriction enzymes were purchased from Takara (TaKaRa Bio Group).

### Generation of in‐frame gene deletion, gene complementation, and promoter‐swapped mutants

The upstream and downstream fragments of the target gene were amplified to construct vectors for in‐frame gene deletion in *P. aeruginosa* PAO1, and the fragments were connected to a linearized pEX18 plasmid to construct the recombinant plasmid pEX18‐tg (target gene)^13^. The recombinant plasmid pEX18‐tg was transformed into competent *E. coli* DH5*α* cells by heat shock transformation. The transformants were selected with 25 μg/ml gentamicin and verified by agarose gel electrophoresis, and then, the *E. coli* DH5*α* transformant DH5*α*‐pEX18‐tg was obtained. The *E. coli* S17‐1‐pEX18‐tg transformants and *P. aeruginosa* PAO1 were mixed for conjugate transformation and selected by kanamycin and gentamicin. Clones were transferred to a plate containing 10% sucrose and 50 μg/ml kanamycin to obtain the second homologous recombination strain. The mutant was then verified by PCR. The target gene was ligated to the linearized pBBr1 MCS‐5 plasmid by enzyme digestion to construct the gene complement vector pBBr1 McS‐5‐tg. This vector was introduced into the mutant (Δtg) by electrotransformation. The transformants were selected by gentamicin and verified by PCR. The gene complementation strain Δtg::tg was obtained. The vector used in the promoter replacement experiment was pEX18GM. *groEL* promoter replacement in *P. aeruginosa* PAO1 was realized by connecting upstream and downstream homology arms. Details of the primers, restriction sites, and fragment lengths are shown in Table S3. All of the mutants were verified by PCR and Sanger sequencing (Figures S12–14).

### Survival rate assay

The wild‐type PAO1 and mutant strain Δ*lasR* were incubated in Luria‐Bertani (LB) medium for 12 h until OD_600_ = 1, 1 ml of suspension was centrifuged, and the supernatant was discarded. Then, the experiments were designed separately according to different adversity conditions and resuspended using 3 ml of the corresponding medium. An antioxidant capacity assay was performed at a final concentration of 2 mM H_2_O_2_, with different concentrations of MomL added to the experimental and control groups, and the number of colonies was compared by gradient dilution of coated plates after standing at 30°C for 6 h.

### Biofilm assay

The wild‐type PAO1 (5 × 10^5^ colony‐forming units [CFU]/ml) was cultured in 10% TSB (with or without 0.1 U/ml MomL) for 18 h in a 96‐well plate. Three parallel experiments were performed. All groups were washed, and 100 μl of methanol was added to fix them for 20 min. Then, 100 μl of 1% crystal violet solution was added to dye them for 20 min, and they were then washed again. After they were treated with 80 μl of 95% ethanol for 30 min, the value of OD_570_ of them were measured.

### Pyocyanin extraction and determination assay

The bacteria were injected in Pseudomonas Agar Medium for Detection of Pyocyanin (PDP) medium for 16‐18 h. PDP without bacteria was the negative control. The cultures were centrifuged at 8000*g* for 10 min, and then, chloroform was added to the supernatant at a ratio of 5:3 for vortex extraction. The mixture was centrifuged at 13,000*g* for 2 min, the chloroform layer was collected, 200 μl of 0.2 M hydrochloric acid was added, and the mixture was vortexed and centrifuged at 8000*g* for 10 min. Then, 200 μl of the upper layer was centrifuged at 8000*g* for 10 min, and the OD_520_ value was obtained in a 96‐well plate with triplicate samples.

### Measurement of enzyme kinetic parameters

A 2× 3‐Morpholinopropanesulfonic Acid storage solution (MOPS, 5 mM, pH 7.1) and 10× Bromothymol blue storage solution (BTB, 1 mM) were prepared. A total of 50 μl of MOPS stock solution, 10 μl of BTB stock solution, 0–250 mM AHLs, and dimethyl sulfoxide at a 1% final concentration were combined. The appropriate amount of recombinant MomL to be tested was added, and a final volume of 100 μl was achieved with triple‐distilled water at 25°C. The mixture was analyzed 50 times continuously (every 30 s) at 630 nm with an enzyme marker. The initial reaction speed of each concentration of AHLs was calculated and converted according to the standard curve. Triplicate experiments were conducted for each experimental group.

### Transcriptional profiling and analysis

Transcriptional profiling of *P. aeruginosa* PAO1 (in the absence or presence of 0.1 U/ml MomL) was carried out by the Biozeron Company (PRJNA625005). Total RNA of *P. aeruginosa* PAO1 (with and without 0.1 U/ml MomL) was extracted with TRIzol reagent (Invitrogen). RNA quality was quantified using a Bioanalyzer 2100 (Agilent) and NanoDrop 2000. RNA transcript libraries were constructed by a TruSeq RNA Library Preparation Kit of Illumina. Library sequencing was performed on the Illumina HiSeq platform. EdgeR^32^ was used for differential gene expression analysis (https://bioconductor.org/packages/-release/bioc/html/edgeR.html). Clean reads were aligned to the reference genome using Rockhopper (http://cs.wellesley.edu/%7Ebtjaden/Rockhopper/). Gene Ontology functional enrichment and Kyoto Encyclopedia of Genes and Genomes pathway analyses were carried out by Goatools (https://github.com/tanghaibao/Goatools) and KOBAS, respectively (http://kobas.cbi.pku.edu.cn/home.do). Differences greater than two folds with a *p* < 0.005 were regarded as significant differences.

### Fluorescent antibiotic preparation and cell staining for microscopy

Fluorescently labeled kanamycin (C_18_H_38_N_4_O_15_S; MW = 582.58) was added to anhydrous dimethylformamide (DMF), and then triethylamine was added to the reaction system. 5(6)‐carboxyfluorescein diacetate, succinimidyl ester (CFDA‐SE), was dissolved in DMF and added to the kanamycin solution for a 4 h reaction. Thin‐layer chromatography detection and high‐performance liquid chromatography purification were performed. Mass spectrometry was used to verify the chemical structure of kanamycin‐CFDA. For kanamycin‐CFDA staining, the final concentration was 50 μg/ml. The cells were incubated in the dark with slow shaking at 30°C for 6 h. The cells were collected and washed three times with LB medium, and then observed under a laser scanning confocal microscope.

### Phospholipase A (PldA) assay

Phospholipase A (PldA) can react with 2‐thiohexadecylethyl phosphate choline (HEPC) to produce free sulfhydryl groups and with 5,5′‐dithiobis‐(2‐nitrobenzoic acid) to produce a yellow substance with a characteristic absorption peak at 412 nm. In this study, a PldA test kit (MS2414) was used to detect the activity of PldA. The bacterial solution cultured for 18 h (100 μl) was added to 5 ml of extract solution and the supernatant was collected after sonication. The supernatant was discarded by centrifugation, and the precipitate was retained and dissolved in 1 ml of reagent as the sample. The control and the treatment were set up for each group of samples. Then, 20 μl of the samples was added to each well of sterile 96‐well plates. In the control and the treatment, 180 μl of reagent 2 and reagent 3, respectively, was added, and the mixture was then blended, mixed, and incubated at 37°C for 10 min. A412 was detected by an enzyme marker. PldA enzyme activity was defined as the amount of enzyme required to hydrolyze HEPCs to produce 1 nM of free sulfhydryl groups per minute per 10^4^ cells per unit of enzyme activity. ΔA = A (treatment) − A (control). PldA activity (nmol/min/10^4^ cell) = 73.53 × ΔA ÷ cell number.

### MIC assay

The MIC of antibiotics combined with MomL for *P. aeruginosa* PAO1 was determined using a two‐fold dilution technique in 96‐well microtiter plates, as described by the Clinical and Laboratory Standards Institute guidelines. In this assay, *P. aeruginosa* PAO1 was grown in Mueller‐Hinton broth and prepared as a bacterial suspension for use. Antibiotics with an original concentration of 1024 μg/ml were used to make two‐fold dilutions. MomL (0.1 or 0.5 U/ml) was added to the corresponding wells, and 5 µl of diluted bacterial suspension was added to each well (to obtain a final concentration of *ca*. 5 × 10^5^ CFU/ml). The MIC assays of MomL, kanamycin, and tobramycin were also conducted with the two‐fold dilution. Antibiotics with original concentrations of 1024 and 1000 μg/ml were used to make dilutions and then cocultured with bacterial suspensions. To determine the effective concentration of the MomL–kanamycin combination, different concentrations of MomL were combined with 64, 32, and 16 μg/ml kanamycin. Then, 10 µl of the diluted bacterial suspension was added to each well and incubated at 37°C for 18 h, and the results were recorded. The measurements were performed in triplicate.

### Pathogenicity assay

Zebrafish larvae were bred in a pure culture system, MomL (0.8 μg/ml) was added at the 5th hour, 10^8^ CFU/ml *P. aeruginosa* PAO1 was added at the 6th hour, the bacterial solution was washed off, and fresh medium and MomL were added at the 8th hour. The death of fish was continuously observed, and the survival rate was counted for 96 h.

### Heterologous expression and purification of proteins

The method described by Tang et al.^33^ was applied with some modifications. The gene fragments were obtained by PCR using *P. aeruginosa* PAO1 genomic DNA as a template. The purified PCR amplification products and pET28a plasmids were double‐digested, and the digested products were ligated at 16°C for 16 h. The ligation products were transferred into *E. coli* BL21 (DE3) competent cells by heat shock. The expression strains were obtained, and then, they were cultured in the LB liquid medium containing 50 μg/ml kanamycin at 37°C. Expression was induced with 0.5 mM Isopropyl‐β‐D‐thiogalactopyranoside (IPTG), and the cells were grown at 16°C and 150 rpm for 12 h. The bacterial cells were collected and disrupted by ultrasonication, and the supernatant was collected by centrifugation. A Ni‐NTA affinity chromatography purification system (Qiagen) was used to purify MomL, and 12% SDS–PAGE was used to characterize the protein expression and purification results. Purified proteins (MomL, LasR and RpoS) were dialyzed and concentrated in dialysis bags and ultrafiltration tubes and stored at ‐20°C. For the LasR expression strain, the LB liquid medium contained 20 μM 3‐*O*‐C_12_‐HSL.

### Bioassay to assess the intracellular AHL degradation activity of MomL

*P. aeruginosa* PAO1 was inoculated into 5 ml of LB liquid medium with 0.13 U/ml MomL/protein buffer and cultured at 37°C with shaking for 18 h (170 rpm). Cells were collected by centrifugation at 12,000*g* for 10 min and washed three times with 0.85% saline. Cells were resuspended in 5 ml of 0.85% saline and placed on ice for disruption. Cell lysates were then centrifuged at 12,000*g* for 5 min to collect the supernatant. The supernatant was extracted with ethyl acetate, evaporated to dryness with a rotary evaporation apparatus, and then dissolved in 500 μl of methanol to obtain the intracellular AHLs. The degradation activity of MomL toward the intracellular AHLs of *P. aeruginosa* PAO1 was determined using the *Chromobacterium violaceum* CV026 plate detection method^34^. One milliliter of an overnight culture of CV026 was added to 15 ml of molten semisolid LB agar. After the medium solidified, 50 µl of intracellular AHLs or methanol was added to the culture. The samples were placed at 28°C and observed after overnight cultivation.

### Bacterial competition assay

*P. aeruginosa* PAO1 cells were cocultured with equivalent numbers of NCIMB2033 cells in marine broth 2216 (MB) supplemented with 0.13 U/ml MomL protein or protein buffer (20 mM Tris‐HCl, 0.85% NaCl; pH 8.0) for 48 h in a 28°C incubator. Following incubation, the mixed bacteria were diluted and plated onto marine agar 2216 (MA) plates. The coated plates were incubated at 28°C for 48 h to observe the results. The visible white and red colonies provided a qualitative indication of *P. aeruginosa* PAO1 and NCIMB2033 survival.

*E. coli* DH5*α* (pUCm‐T) is an engineered strain that carries a pUCm‐T plasmid to allow α‐complementation of β‐galactosidase. *P. aeruginosa* PAO1 cells were cocultured with equivalent numbers of *E. coli* DH5*α* (pUCm‐T) cells in LB broth supplemented with 10 μg/ml ampicillin and 0.13 U/ml MomL protein or protein buffer for 48 h in a 37°C incubator. Following incubation, the mixed bacteria were diluted and plated onto LB agar plates supplemented with 100 μg/ml X‐gal, 24 μg/ml IPTG, and 10 μg/ml ampicillin. The plates were incubated for 36 h at 37°C. The number of blue colonies (*E. coli* DH5*α* [pUCm‐T]) and green colonies (*P. aeruginosa* PAO1) visible on the plate indicated the survival rates of the two strains.

### Electrophoretic mobility shift assay (EMSA)

EMSA in this study was based on the following method. Taking the EMSA between LasR and the promoter of *relA* as an example, the method is presented. A biotin‐labeled promoter‐binding sequence probe of the *relA* gene in *P. aeruginosa* PAO1 was synthesized by Shanghai Shengong Biological Company. The following formula was used to prepare the EMSA gel: 10× TBE buffer (1 ml), ddH_2_O (16.2 ml), 30% polyacrylamide (2 ml), 80% glycerol (625 μl), 10% ammonium persulfate (150 μl), and N,N,N',N'‐Tetramethylethylenediamine (TEMED,10 μl). The negative control group, sample reaction group, cold probe competitive reaction group, mutation cold probe competitive reaction group, and supershift reaction group were set up, and the protein‐probe complexes were formed at 37°C for 30 min. The loading buffer was added and mixed with the sample before loading, followed by electrophoresis at 100 V until the blue dye ran 3/4 of the way down the gel. After removing the EMSA gel, the proteins were transferred to a membrane by the wet transfer method at 380 mA for 60 min, and then ultraviolet (UV) crosslinking was performed immediately. The membrane was washed with buffer solution, and the blocking solution was added for 20 min to seal the membrane. The blocking solution was removed, and an appropriate amount of horseradish peroxidase‐conjugated streptavidin was added for incubation with the membrane at room temperature for 45 min. Subsequently, the membrane was washed three times with a buffer solution. The reaction substrate was evenly added to the membrane. After incubation at room temperature for 5 min, exposure‐based imaging on a chemiluminescence detection system was performed. According to the above method, EMSA between RpoS and the promoter of H2‐T6SS was performed.

### Continuous repeated transfer assay

*P. aeruginosa* PAO1 was inoculated into the medium with 2 μg/ml MomL, and kanamycin was added to the next generation when the bacterial culture reached the stationary phase. The concentration of kanamycin was increased by 2 μg/ml for each bacterial generation. To study the resistance of *P. aeruginosa* PAO1 to kanamycin in the evolutionary process in the presence of MomL, the MIC value for bacteria was compared with that for the primary *P. aeruginosa* PAO1 culture as a control.

### RpoS/H2‐T6SS promoter model building

A homology model of RpoS (RNA polymerase sigma factor) was built in Modeller as described previously^35^. The crystal structure of *E. coli* RpoS was selected as the template (PDB Code: 5IPL) due to its high sequence identity with the RpoS from this study. The model with the highest doping score was selected for DNA docking. The crystal structure of the −35 element DNA (PDB Code: 6JHE) was extracted from the complex and docked to RpoS using HDOCK with the default parameter^36^. The 10 models with the top binding energy ranking scores were selected for the analysis. The surface electrostatic potential was calculated using the APBS module in PyMOL (https://pymol.org/2/).

### Surface plasmon resonance (SPR) assay

The kinetics and specificity of the binding reactions between the RpoS protein and DNA were carried out with the PlexArray®HT SPR system. Briefly, DNA (10 mM) was immobilized on Graft‐to‐PCL sensor chips by UV crosslinking for 15 min. The mobile phase was a solution of RpoS (dissolved in PBS), and the concentrations were set as 0.5, 1, 5, 10, and 20 mM. The data obtained were analyzed and fitted by the PLEXERA SPR Date Analysis Module to obtain the equilibrium dissociation constant (KD).

### Cell viability assay

Human corneal epithelial cells (HCECs) were obtained from American Type Culture Collection (ATCC) and cultured in Dulbecco's modified Eagle's medium Nutrient Mixture F‐12 (Gibco BRL) with 10% fetal bovine serum (Gibco), 100 U/ml penicillin, and 100 μg/ml streptomycin (Gibco). Cells were grown to confluency in 25 cm^2^ polystyrene tissue culture flasks at 37°C in 5% CO_2_ and 95% air, and confluent cells were subcultured every 2–3 days by trypsinization with trypsin/EDTA solution.

To observe the effects of MomL and MomL‐kanamycin on the morphology and proliferation of HCECs, kanamycin was first dissolved in 1.8 U/ml (1250 μg/ml) MomL at a concentration of 640 μg/ml, and then, HCECs were seeded in 96‐well tissue plates (BD) and randomly divided into the following four groups: the control group, 1.8 U/ml (1250 μg/ml) MomL group, 640 μg/ml kanamycin group, and 640 μg/ml kanamycin combined with 1.8 U/ml (1250 μg/ml) MomL group. After 12 and 24 h, the proliferation of HCECs was quantitatively determined by the Cell Counting Kit‐8 assay at an OD value of 450 nm with a microplate reader (BioTek Instruments). The biological toxicity of MomL to cells was similar to those described above. MomL was dissolved at concentrations of 0.5, 1, 1.5, 2, 2.5, and 3 mg/ml, and was cocultured with HCECs for 12 and 24 h. Then, the same method mentioned above was used to measure cell activity.

### Therapeutic effects of MomL‐kanamycin in a mouse model of *P. aeruginosa* PAO1 infection

C57BL/6J mice (6–8 weeks old) were purchased from Beijing Vital River Laboratory Animal Technology Co., Ltd. The mice were housed in an environment with a cycle of 12 h of light and 12 h of dark at 20°C. No disease was found in these animals by slit‐lamp examination or indirect fundoscopy. Briefly, the mice were anesthetized by intraperitoneal injection with pelltobarbitalum natricum (0.3%, 0.1–0.2 ml/10 g). Proparacaine hydrochloride (0.5%) was used topically for corneal anesthesia. When there was no response to corneal touching, three 1‐mm scratches were made in the corneal epithelium with a 25‐gauge needle, and then, inoculation was performed with 2.0 × 10^6^ CFU of *P. aeruginosa* PAO1 in the right eye to establish the PAO1 mouse model. Twenty‐four hours later, these mice were divided into 10 groups after checking the uniformity: the control, MomL (1250 μg/ml), kanamycin (640 μg/ml), kanamycin (640 μg/ml) + MomL (1250 μg/ml), cefazolin (640 μg/ml), cefazolin (640 μg/ml) + MomL (1250 μg/ml), neomycin (640 μg/ml), neomycin (640 μg/ml) + MomL (1250 μg/ml), tobramycin (40 μg/ml), and tobramycin (40 μg/ml) + MomL (1250 μg/ml) treatments. The drugs were subconjunctivally injected each day. At 1, 3, and 5 days after the intervention, the mice were photographed and clinically scored after they were humanely euthanized. The eyes were enucleated and processed for histological examination and quantitative microbial cultivation. All mice were treated in accordance with the guidelines of the Council for Purpose of Control and Supervision of Experiments on Animals, Ministry of Public Health, China.

### Clinical scoring

The inoculated eyes were scored with front segment photography by the slit lamp at 1, 3, and 5 days after subconjunctival injection. Clinical scores were designated as follows: “0” represents no opacity; “+1” represents slight opacity with iris unobscured; “+2” represents dense opacity with iris partly unobscured; “+3” represents dense opacity with iris fully unobscured; and “+4” represents corneal perforation or phthisis.

### Histopathological examinations

Infected eyes were enucleated from euthanized mice, fixed in 4% paraformaldehyde, and then embedded in paraffin 1, 3, and 5 days after the operation. Continuous 5 μm sections were stained with hematoxylin‐eosin (HE). The histological structure and the degree of inflammation were evaluated by light microscopy. Then, the quantitative evaluation of inflammatory cells and corneal edema was evaluated by MATLAB 2017b and ImageJ. Specifically, the HE‐stained corneal images were converted into binary images and analyzed by ImageJ. The number of inflammatory cells was measured by FreeHand selection. For corneal edema, the measurement area was calibrated with the FreeHand Selection tool, and then the measurement parameters were set in Analyse‐Set Measurements, which used the line tool to measure the thickness of the cornea.

### Quantification of viable bacteria

Whole corneas from the murine model of *P. aeruginosa* PAO1 infection were placed in 1 ml of sterile saline (0.85% NaCl, pH 7.4) containing 0.25% bovine serum albumin (BSA) and homogenized. Serial 10‐fold dilutions were prepared and plated in triplicate on a selective culture medium (Difco Pseudomonas Isolation Agar; BD Biosciences, Inc.). The plates were then incubated at 37°C for 18–24 h, and the CFU number was determined by direct counting.

### Statistical analysis

Statistical analyses were performed using SPSS version 21.0 (IBM). Differences in the clinical scores, bacterial viability, and inflammatory cells were analyzed via one‐way analysis of variance with the least significant difference post hoc test. Significant differences were defined as *p *< 0.05.

## AUTHOR CONTRIBUTIONS

Yan Wang, Jin Yuan, Xiao‐Hua Zhang conceived the project. Yan Wang designed the experiments. Jin Yuan, Qin Dou, Rilei Yu, Jiahui Yang, Jiayi Wang, Yuxiang Zhu, Yuying Li, Yichen Xiao, and Jiazhen Liang carried out the experiments. Yan Wang, Rilei Yu, and Xiao‐Hua Zhang analyzed the data. Yan Wang wrote the manuscript draft. Jin Yuan, Xiao‐Hua Zhang, Jing Zhong, Rilei Yu, Hongan Long, and Yuxiang Zhu revised the manuscript. All the authors have read and approved the submission for publication.

## ETHICS STATEMENT

The animal experiments conformed to the Association for Research in Vision and Ophthalmology Statement for the Use of Animals in Ophthalmic and Vision Research. The research protocol was also approved by the Animal Care Committee of Zhongshan Ophthalmic Center, Sun Yat‐sen University (Guangzhou, China) (approval ID: 2020011).

## CONFLICT OF INTERESTS

The authors declare no conflicts of interests.

## Supporting information

Supporting information.

## Data Availability

The datasets generated and/or analyzed during the current study are available from the corresponding authors on reasonable request.
