# Supplementary material for: MomL inhibits bacterial antibiotic resistance through the starvation stringent response pathway
Source: mLife. 2022 Mar 24;1(4):428–42. doi: 10.1002/mlf2.12016 (PMC10989899; doi:10.1002/mlf2.12016)
Supplement: Supplementary file 1 — Supporting information. [file MLF2-1-428-s001.docx]

**MomL inhibits bacterial antibiotic resistance through the starvation stringent response pathway**

**MomL inhibits bacterial antibiotic resistance**

Qin Dou^1,†^, Jin Yuan^2,†^*, Rilei Yu^3,†^, Jiahui Yang^2,†^, Jiayi Wang^1^, Yuxiang Zhu^1^, Jing Zhong^2^, Hongan Long^1^, Zhiqing Liu^1^, Xianghong Wang^1^, Yuying Li^1^, Yichen Xiao^2^, Jiazhen Liang^3^, Xiao-Hua Zhang^1,4,^*, Yan Wang^1,4^*

^1^ College of Marine Life Sciences, and Institute of Evolution & Marine Biodiversity, Ocean University of China, Qingdao 266003, China

^2^ State Key Laboratory of Ophthalmology, Zhongshan Ophthalmic Center, Sun Yat-Sen University, Guangzhou 510064, China

^3^ Key Laboratory of Marine Drugs, Chinese Ministry of Education, School of Medicine and Pharmacy, Ocean University of China, Qingdao, 266003, China

^4^ Laboratory for Marine Ecology and Environmental Science, Qingdao National Laboratory for Marine Science and Technology, Qingdao 266071, China

^†^These four authors contributed equally.

^*^Correspondence: [wangy12@ouc.edu.cn](mailto:wangy12@ouc.edu.cn); [xhzhang@ouc.edu.cn](mailto:xhzhang@ouc.edu.cn); [yuanjincornea@126.com](mailto:yuanjincornea@126.com)

ORCID: 0000-0003-4632-5786


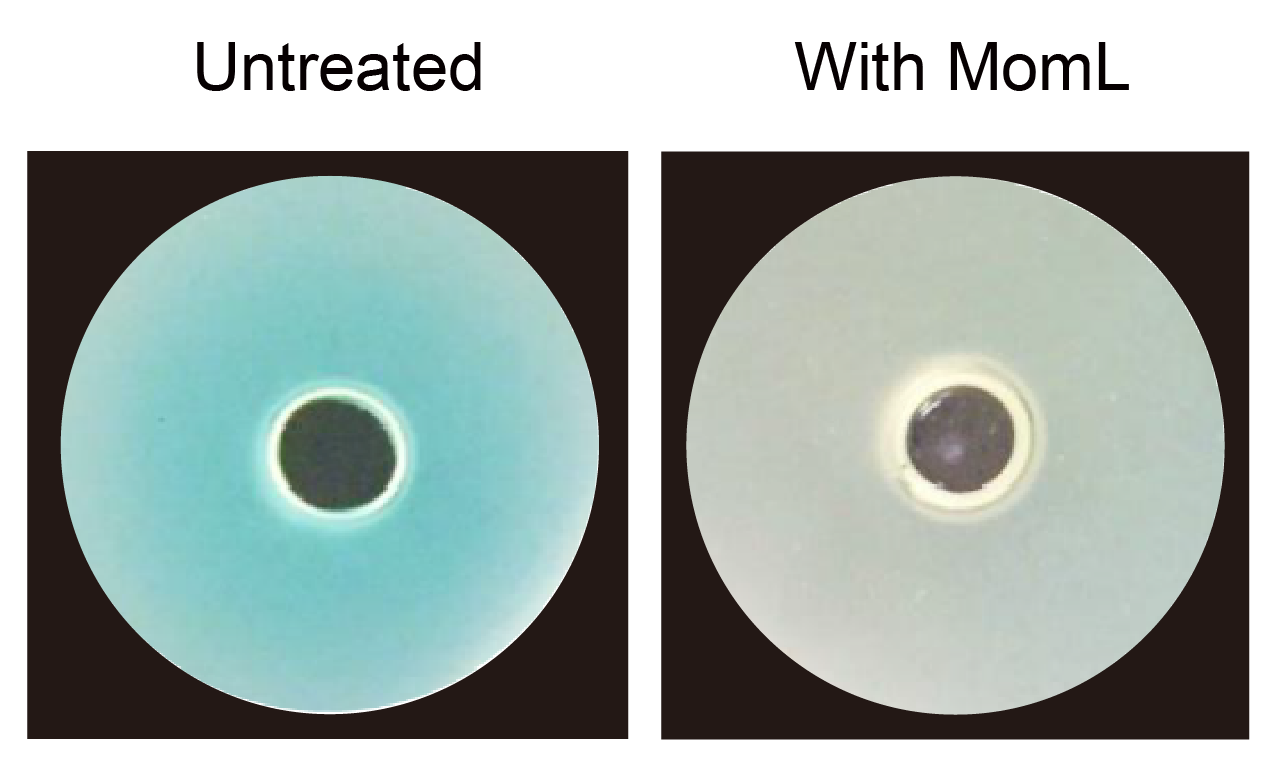


**Supplementary Figure 1. The CV026 plate showed the ability of MomL to degrade extracellular AHLs of *P. aeruginosa* PAO1.** In the untreated group, the interaction between CV026 and the signaling molecules, AHLs, led to the blue plate. In the MomL treatment group, AHLs were degraded by MomL so that the interaction disappeared.


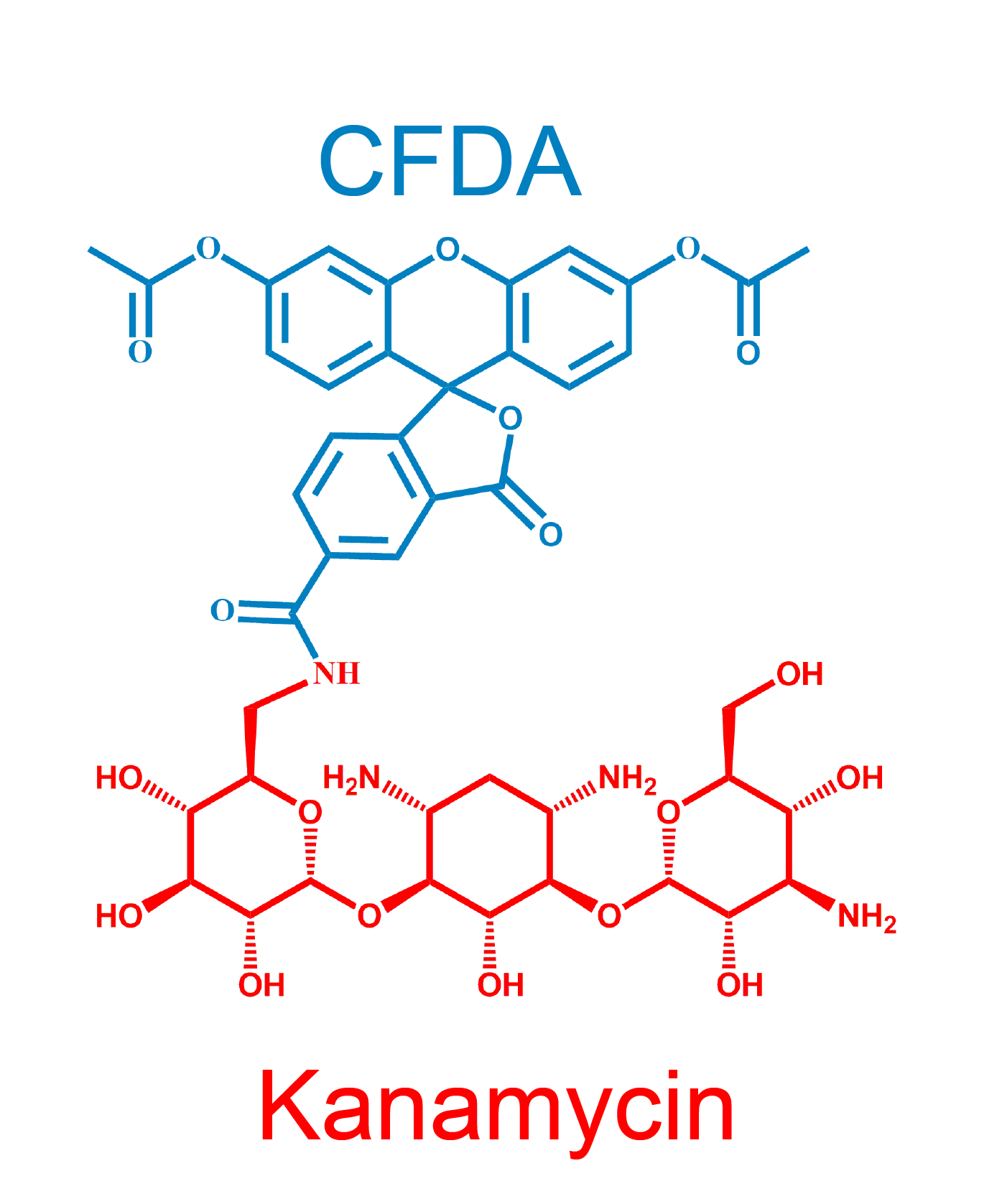


**Supplementary Figure 2. The structure of fluorescent labeled kanamycin.**


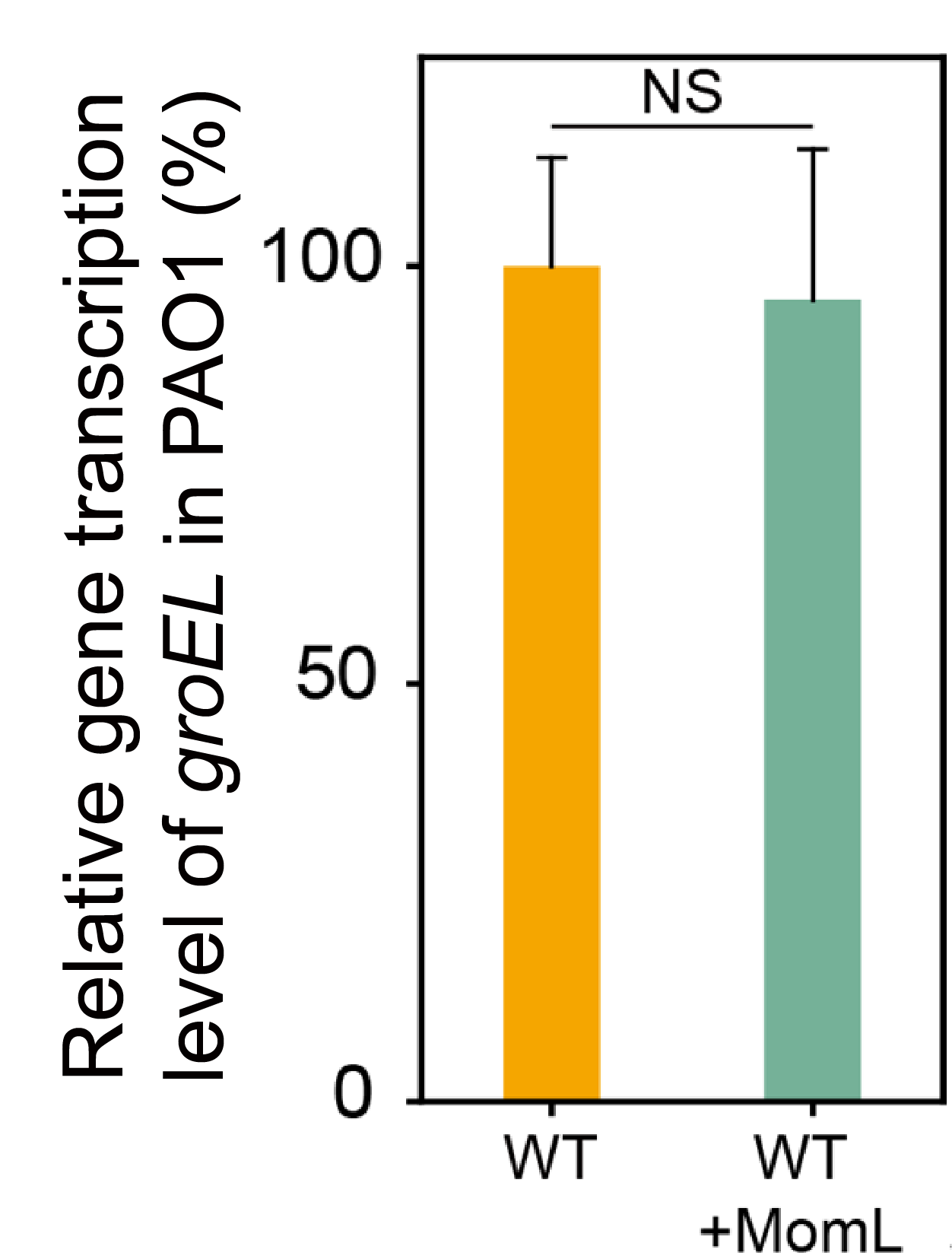


**Supplementary Figure 3. The transcription level of *groEL* in *P. aeruginosa* PAO1.** The expression of *groEL* was unaffected with MomL. Error bars show the standard deviation of three replicates. NS, not significant; **p* value < 0.05; ***p* value < 0.01; ****p* value < 0.001. All data are mean ± s.e.m.


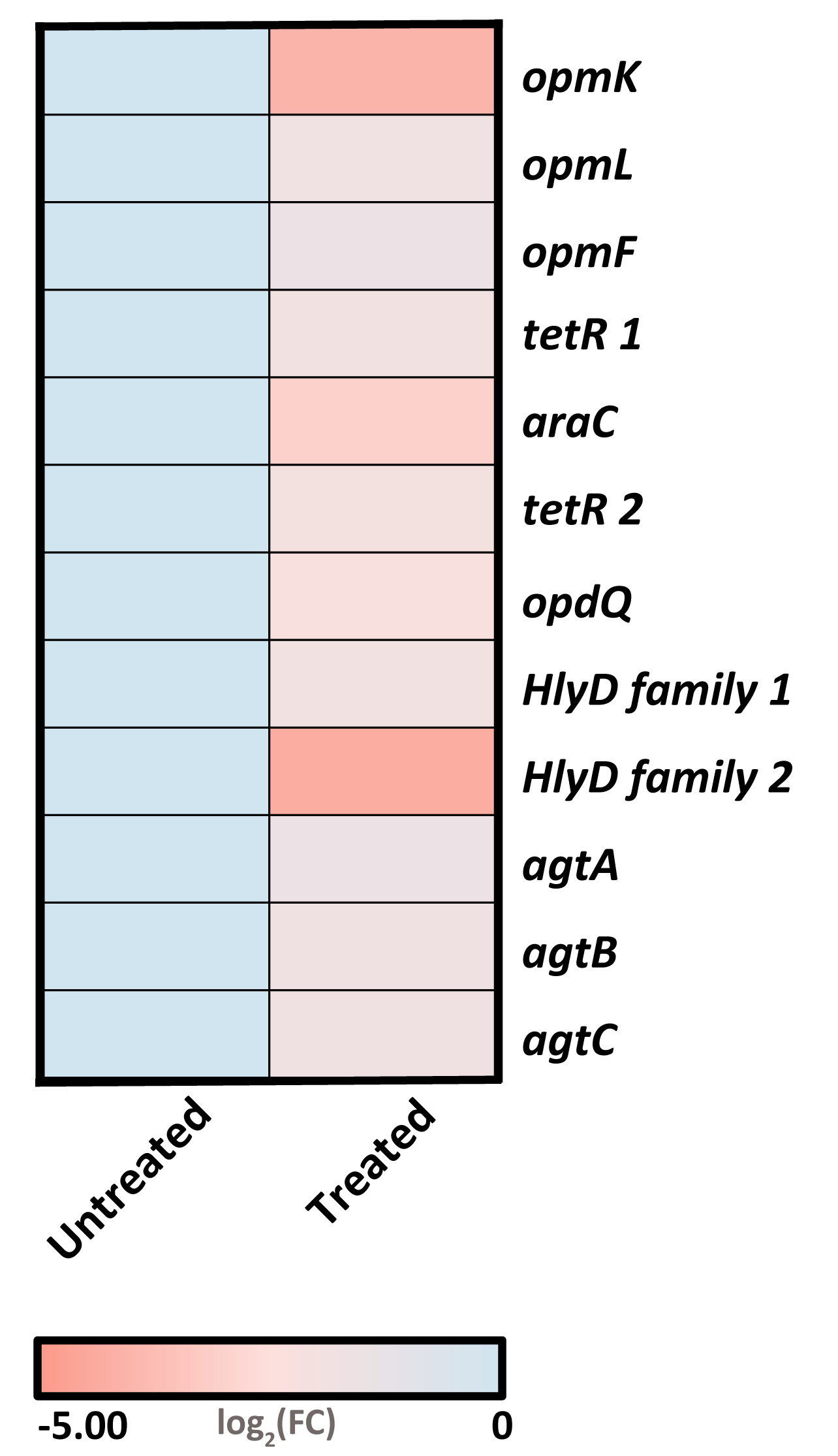


**Supplementary Figure 4. Heatmap showing the relative transcript levels of efflux pump genes regulated by MomL.**


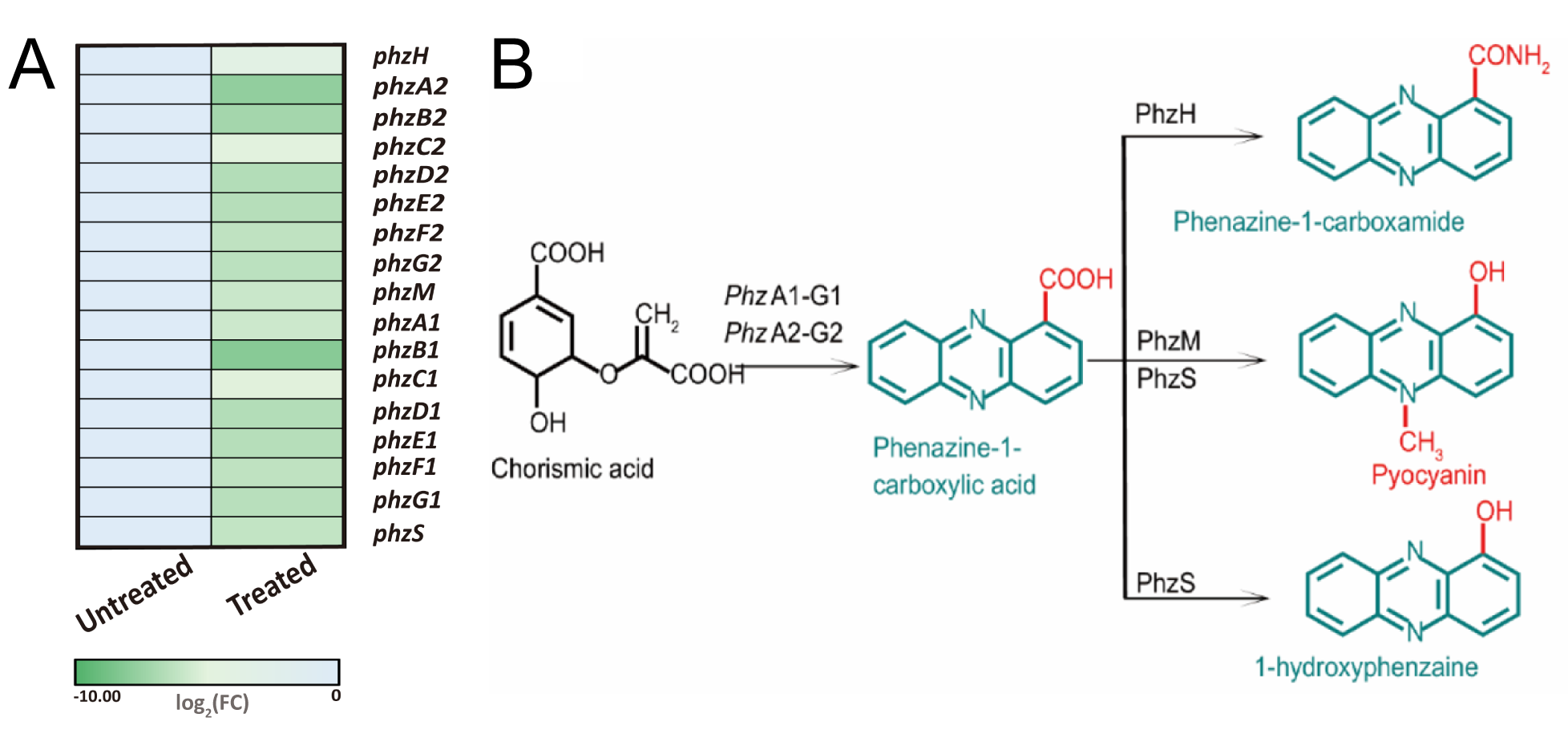


**Supplementary** **Figure 5. Synthetic steps of virulence factors of *P. aeruginosa* PAO1 and relative transcription levels of biosynthesis genes.** **A**, Heatmap showing the relative transcript levels of a cluster of 17 genes involved in the synthesis of phenazines. The scale below the heatmap indicates the fold change in the relative expression level. **B**, Mechanism of phenazine synthesis in *P. aeruginosa* PAO1.


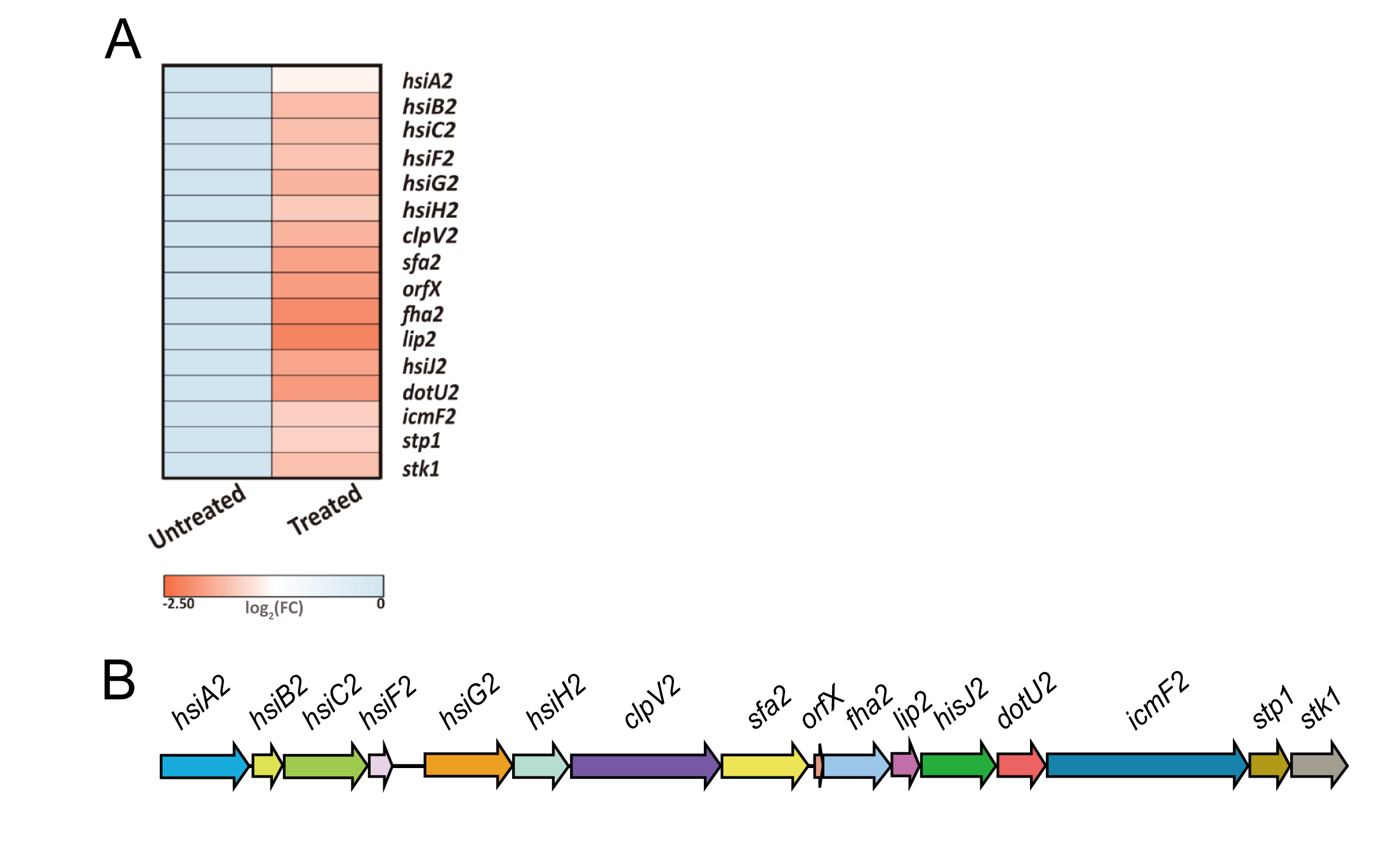


**Supplementary Figure 6. The gene clusters of H2-T6SS and their relative expression levels. A**, Heatmap showing the relative transcript levels of a cluster of 16 genes annotated as the type VI secretion system H2-T6SS. The scale below the heatmap indicates the fold change in the relative expression level. **B**, The H2-T6SS gene cluster of *P. aeruginosa* PAO1, which is annotated as a type VI secretion system.


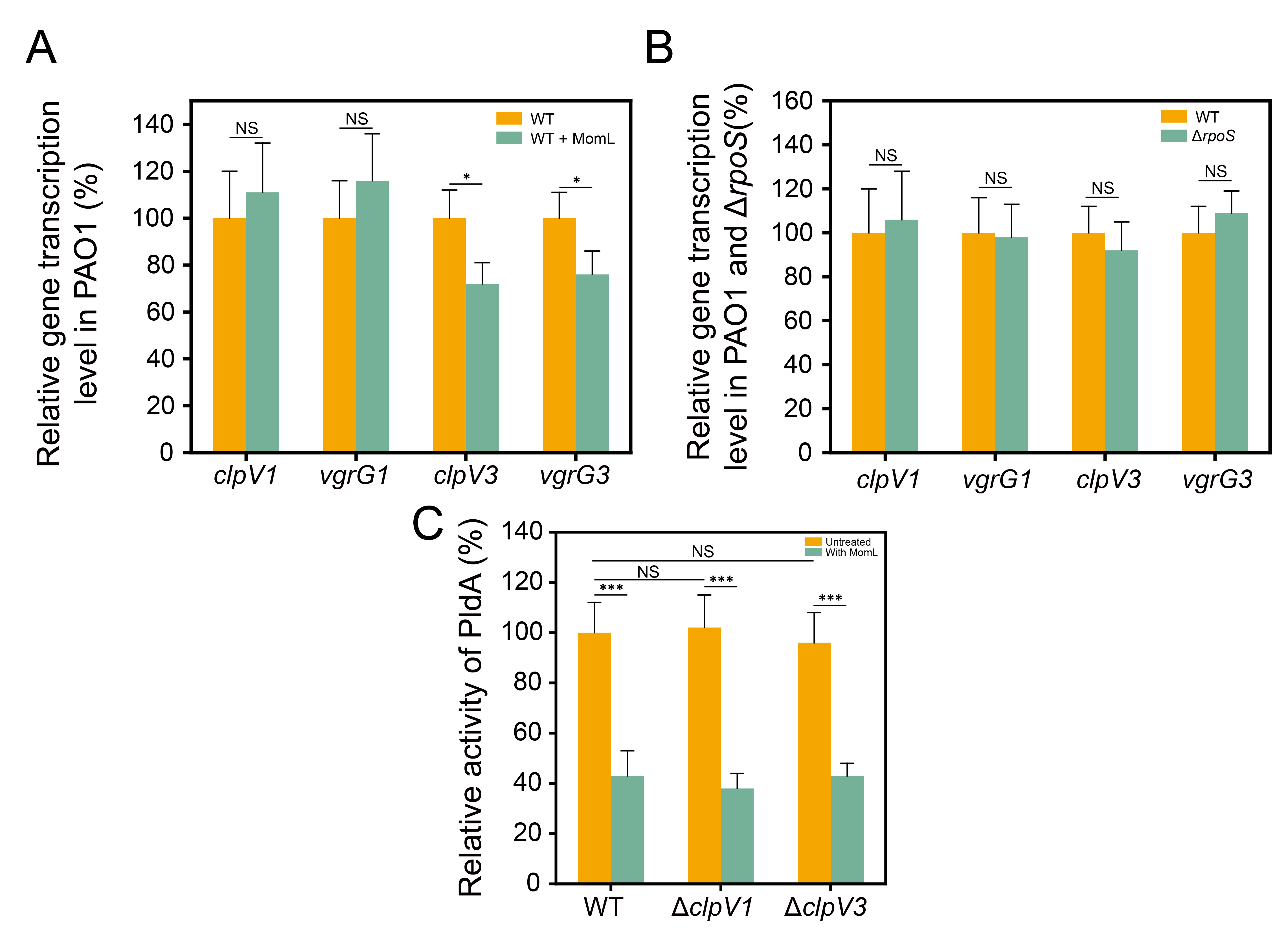


**Supplementary Figure 7. H1-T6SS and H3-T6SS were not involved in this regulatory pathway. A,** Real-time PCR assays of the relative expression levels of the H1-T6SS genes and H3-T6SS genes in *P. aeruginosa* PAO1. The genes *clpV1* and *vgrG1* are related with H1-T6SS. The genes *clpV3* and *vgrG3* are related with H3-T6SS. Under MomL treatment, the transcription level of *clpV1* and *vgrG1* was unaffected with MomL, and the transcription level of *clpV3* and *vgrG3* was downregulated by MomL. **B,** Real-time PCR assays of the relative expression levels of the H1-T6SS genes and H3-T6SS genes in *P. aeruginosa* PAO1 and Δ*rpoS*. The inactivation of *rpoS* could not affect the expression of H1-T6SS genes and H3-T6SS genes. The gene *rpoS* could not be involved in the regulation of H1-T6SS and H3-T6SS. **C,** The relative activity of PldA in the wild-type strain, Δ*clpV1* and Δ*clpV3* under different conditions (with or without MomL). The inactivation of these two genes could not affect the inhibition on PldA activity by MomL. Error bars show the standard deviation of three replicates. NS, not significant; **p* value < 0.05; ***p* value < 0.01; ****p* value < 0.001. All data are mean ± s.e.m.


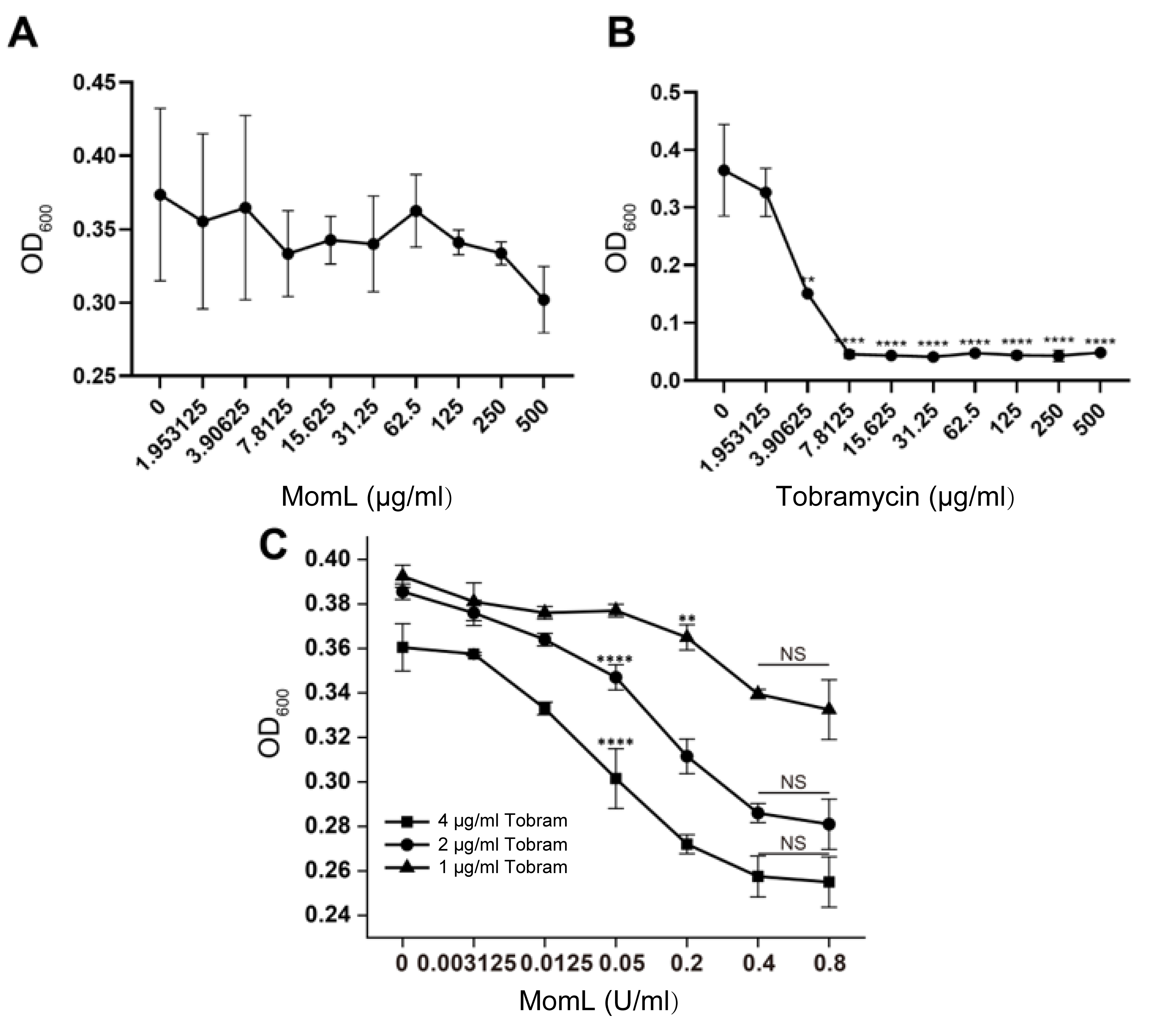


**Supplementary Figure 8. Determining MIC of MomL and tobramycin in different treatment groups.** **A**, **B**, The MIC assay of MomL and tobramycin against *P. aeruginosa*. **C,** The MIC test of MomL with different concentrations of tobramycin (4 μg/ml, 2 μg/ml, 1 μg/ml) against *P. aeruginosa*. Error bars show the standard deviation of three replicates. NS, not significant; **p* value < 0.05; ***p* value < 0.01; ****p* value < 0.001; *****p* value < 0.0001. All data are mean ± s.e.m.


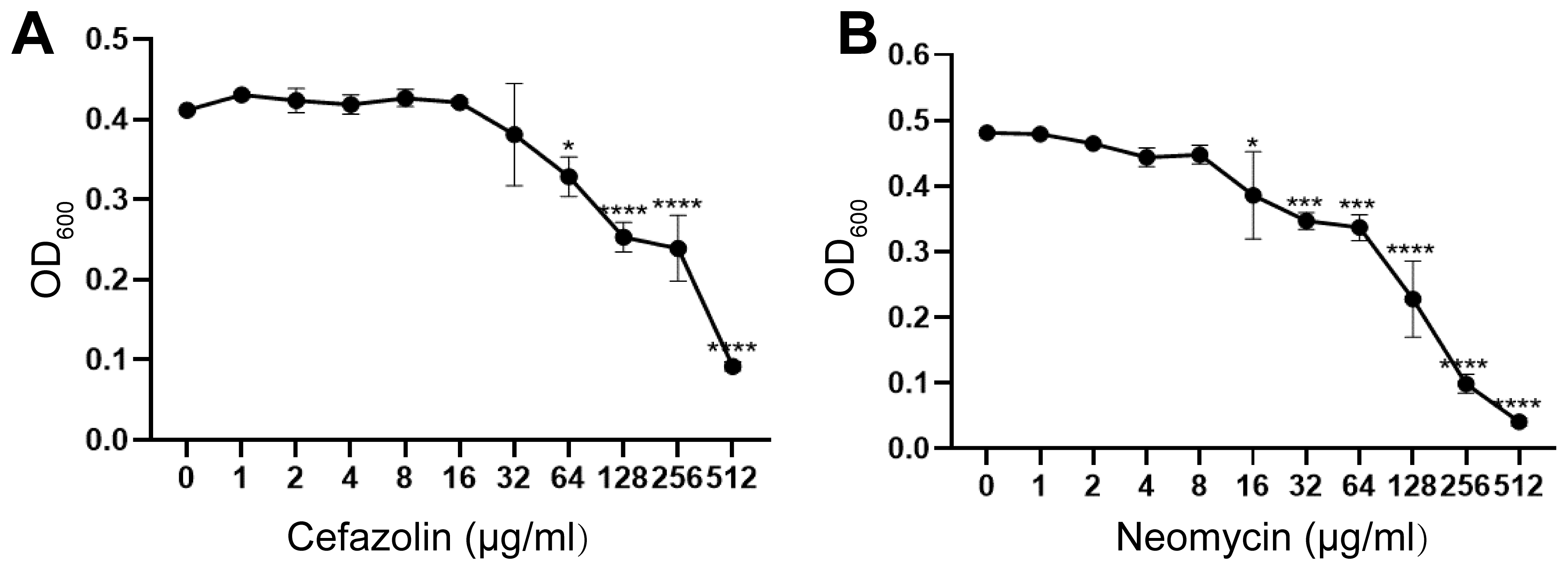


**Supplementary Figure 9. Determining MIC of cefazolin and neomycin in different treatment groups.** **A**, **B**, The MIC assay of cefazolin and neomycin against *P. aeruginosa*. Error bars show the standard deviation of three replicates. NS, not significant; **p* value < 0.05; ***p* value < 0.01; ****p* value < 0.001; *****p* value < 0.0001. All data are mean ± s.e.m.

**Supplementary Figure 10. MomL inhibits *P. aeruginosa* resistance to increase the efficacy of cefazoline and neomycin *in vivo* and *in vitro*.** **A**, **B**, The MIC test of MomL with different concentrations of cefazoline and neomycin (16 μg/ml, 32 μg/ml, 64 μg/ml) against *P. aeruginosa*. **C, D,** Representative ocular pictures and clinical scores in the control, MomL, cefazoline, MomL-cefazoline, neomycin and MomL-neomycin treatment in the *P. aeruginosa* PAO1 animal model at 1, 3 and 5 days. Magnification: 16×. **E,** The histopathology of the control, MomL, cefazoline, MomL-cefazoline, neomycin and MomL-neomycin groups at 1, 3 and 5 days. Magnification: 100×. The blue arrow indicated inflammatory cells infiltrating the corneal tissue. The red arrow indicated inflammatory cells invading the anterior chamber. **F,** The inflammatory cell count of the whole cornea in the HE staining of the control, MomL, cefazoline, MomL-cefazoline, neomycin and MomL-neomycin treatments at 1, 3 and 5 days. **G,** Viable bacterial loads in colony-forming units (CFU) of the control, MomL, cefazoline, MomL-cefazoline, neomycin and MomL-neomycin groups at 1, 3 and 5 days. Error bars show the standard deviation of three replicates. NS, not significant; **p* value < 0.05; ***p* value < 0.01; ****p* value < 0.001. All data are mean ± s.e.m.


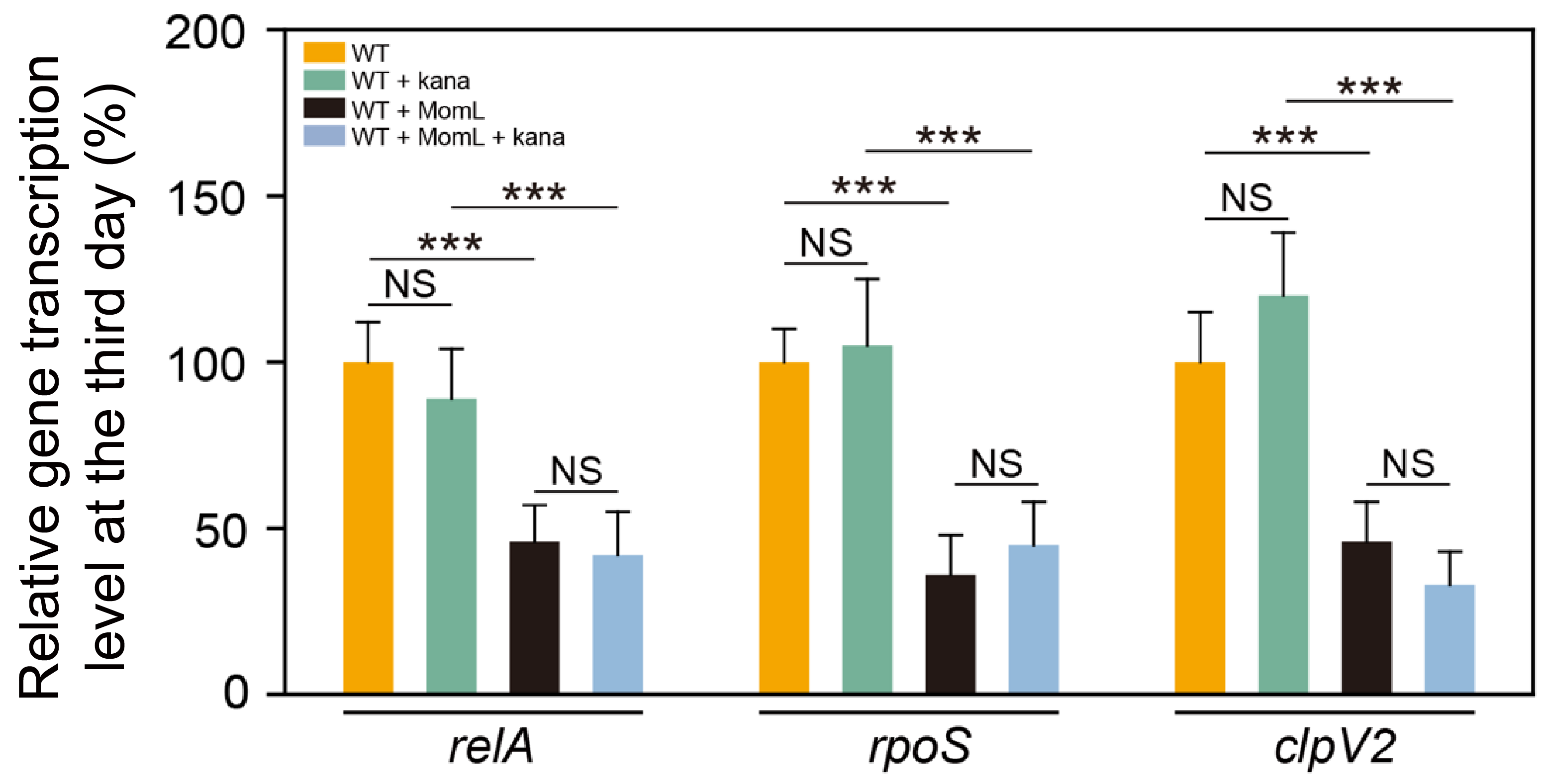


**Supplementary Figure 11. The transcription level assay of *relA*, *rpoS* and *clpV2* during the infection process.** At the third day of the infection process, the transcription levels of these three genes were significantly downregulated by MomL. This effect was unaffected with kanamycin. This result revealed the link between *in vitro* and *in vivo* data. Error bars show the standard deviation of three replicates. NS, not significant; **p* value < 0.05; ***p* value < 0.01; ****p* value < 0.001. All data are mean ± s.e.m.

**
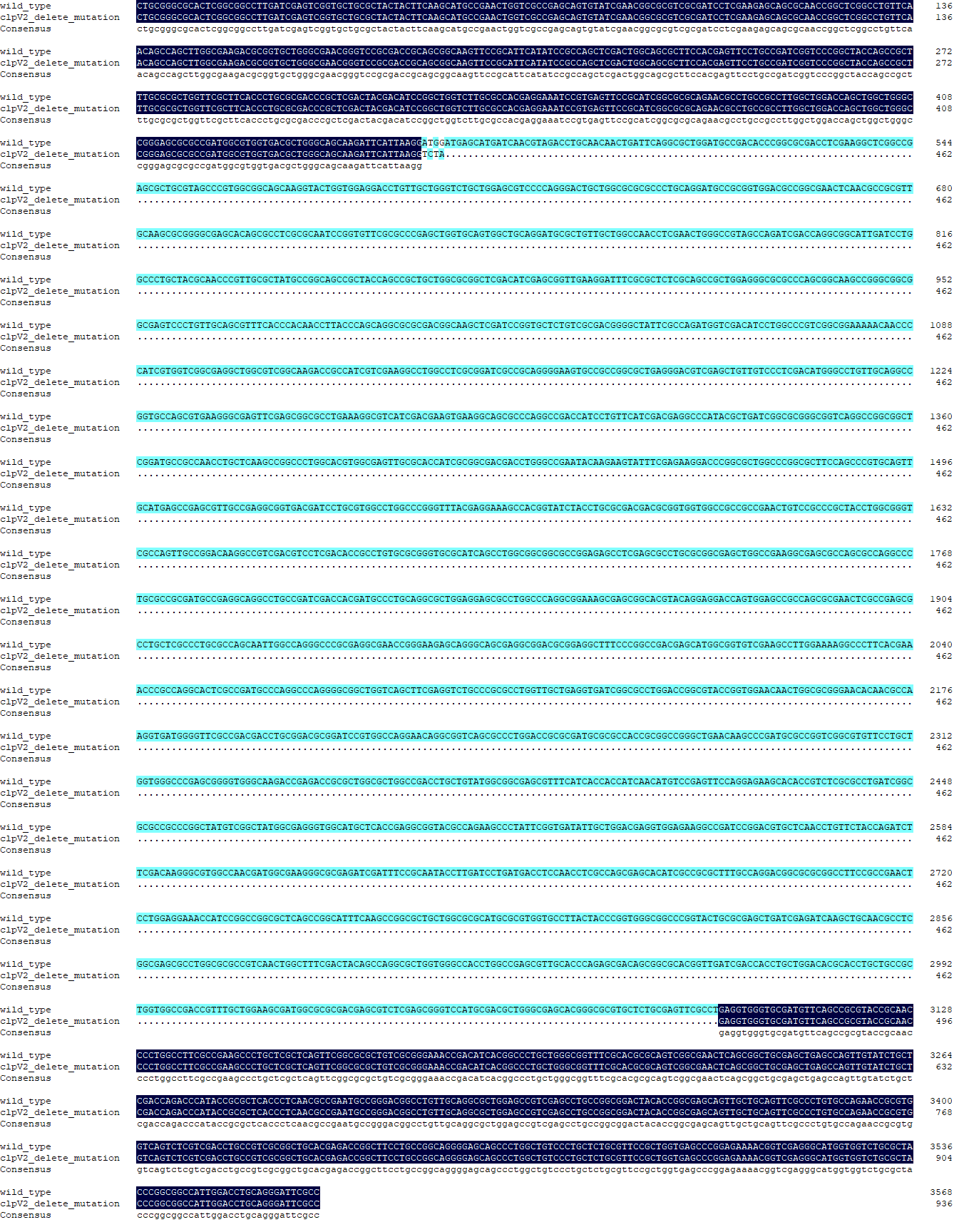
**

**Supplementary Figure 12. Verification of the *clpV2* deletion mutant strain by sequencing.**

**
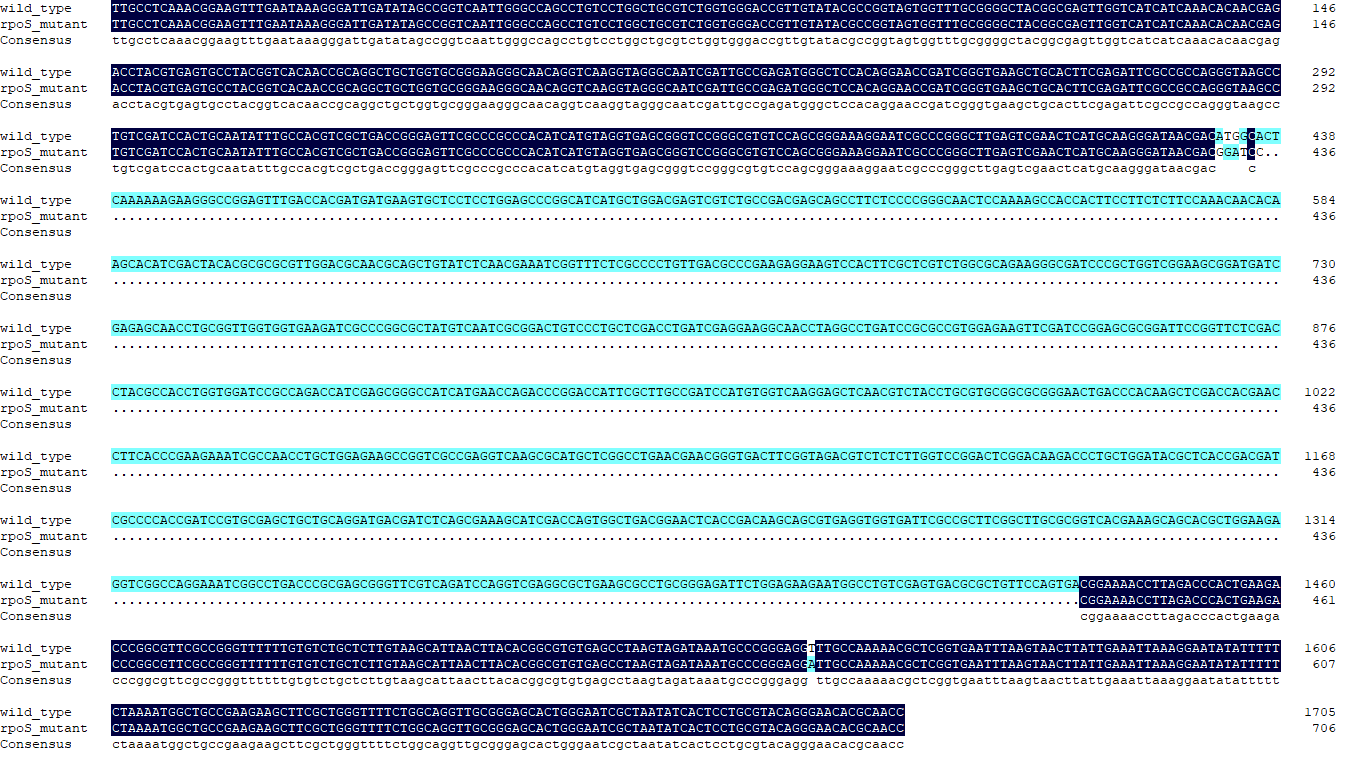
**

**Supplementary Figure 13. Verification of the *rpoS* deletion mutant strain by sequencing.**

**
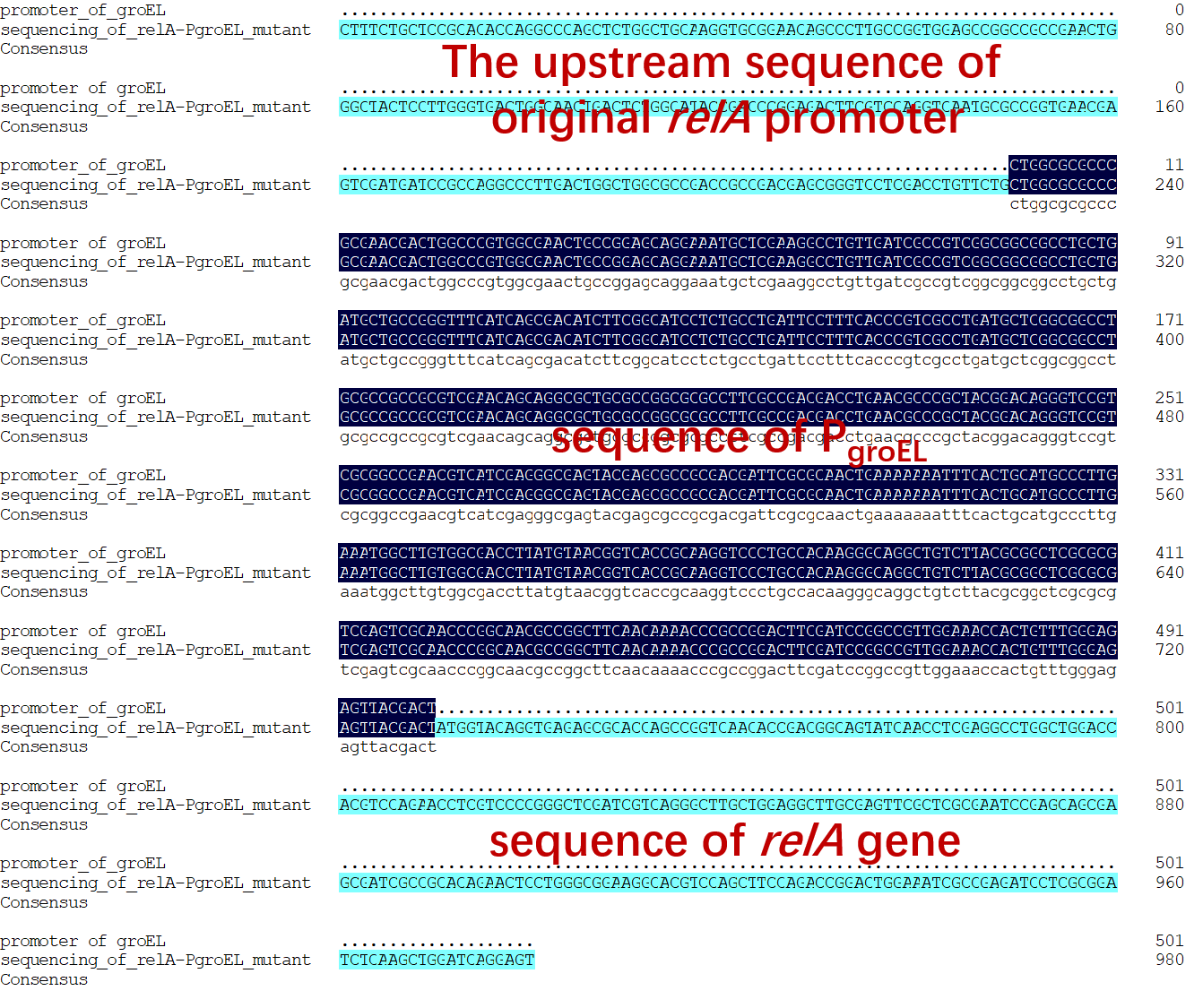
**

**Supplementary Figure 14. Verification of the *relA* promoter-swapped mutant strain by sequencing.**

**Supplementary Table 1.** MIC of other antibiotics on PAO1 in the antibiotic alone group and MomL-treated group.

| **Antibiotic** | MIC (μg/ml) | |
| --- | --- | --- |
|  | Antibiotic alone | With  MomL |
| Gentamicin | 4 | 1 |
| Polymyxin B | 8 | 4 |
| Erythromycin | 16 | 8 |
| Tetracycline | 16 | 8 |

**Supplementary Table 2.** Bacterial strains and plasmids used in this study.

| Strains and plasmids | Relevant characteristics |
| --- | --- |
| Strains | |
| *Pseudomonas aeruginosa* PAO1 | Wild-type |
| *Pseudoalteromonas flavipulchra* NCIMB 2033^T^ | Strain with red color  This strain was used for the bacterial competition assay |
| *Escherichia coli* DH5α carrying the pUCm-T plasmid | Strain with blue color  This strain was used for the bacterial competition assay |
| *E. coli* S17-1 | RP4-2Tc::Mu-Kn :: Tn7 pro hsdR recA;  host for the required plasmids; conjugal donor |
| *E. coli* BL21 | The strain used for heterologous expression of *lasR*. |
| *∆relA*-P*_groEL_* | Mutant strain with a constitutive high-expression promoter P*_groEL_* |
| *∆lasR* | *lasR* gene deletion strain |
| *∆clpV1* | *clpV1* gene deletion strain |
| *∆clpV2* | *clpV2* gene deletion strain |
| *∆clpV3* | *clpV3* gene deletion strain |
| *∆rpoS* | *rpoS* gene deletion strain |
| *∆rpoS*::*rpoS* | The *rpoS* complementary strain of *∆rpoS* |
| *∆clpV2*::*clpV2* | The *clpV2* complementary strain of *∆clpV2* |
| Plasmids | |
| pEX18GM | Gm^r^; *oriT*^+^ *sacB*^+^, gene replacement vector with the MCS from pUC18 |
| pET28a | The plasmid used for the heterologous expression. |
| pBBr1 MCS-5 | This plasmid used for target gene complementation. |
| pHmgA-P-G | The complementary gene was linked to pHmgA-P and formed pHmgA-P-G. This plasmid was used for expression of the target gene. |

**Supplementary Table 3.** Primers used in this study.

| Primers | Sequences | Purpose |
| --- | --- | --- |
| *hsiA2*-up | 5'- GACCTATTCGAGCAAGCTCTCCTCG-3’ | qPCR |
| *hsiA2*-down | 5'- CGCAGGTCCTTGGATTGGGC-3’ |  |
| *hsiB2*-up | 5'- GCCAGCATCGTCGAGCAGGTG-3’ |  |
| *hsiB2*-down | 5'- CAGCACACGGCTACGGGACTCG-3’ |  |
| *hsiC2*-up | 5'- CCGGAACCAGAACGGCGAATACAAC-3’ |  |
| *hsiC2*-down | 5'- CTGCGGCTTGAGCAATTCCTCGATG-3’ |  |
| *hsiF2*-up | 5'- GACTGGATACGGCAGCCTGTTCGAG-3’ |  |
| *hsiF2*-down | 5'- CATGTCGTTGAGATCGGGCAACCCG-3’ |  |
| *hsiG2*-up | 5'- GTGTTCGCTTCGGTTCTGAACG-3’ |  |
| *hsiG2*-down | 5'- CAGGGGCTGCAAACCCATGC-3’ |  |
| *hsiH2*-up | 5'- GACGAGGCGCTCTACGATCAGTTG-3’ |  |
| *hsiH2*-down | 5'- GCACCGAACAAGCCGATCAGGT-3’ |  |
| *clpV2*-up | 5'- CGTTTCATCACCACCATCAACATG-3’ |  |
| *clpV2*-down | 5'- CACGTCCGGATCGGCCTTC-3’ |  |
| *sfa2*-up | 5'- GGCAAGTTCCGCGAAGACC-3’ |  |
| *sfa2*-down | 5'- CGCATTCCACCAATCCCTTC-3’ |  |
| *orfX*-up | 5'- CTGTTCGTCACTGGCCCGCC-3’ |  |
| *orfX*-down | 5'- CTTGCCGCGATTGACCGCCTGC-3’ |  |
| *fha2*-up | 5'- CGATACCGGCAAGAATCCC-3’ |  |
| *fha2*-down | 5'- CGATACCGGCAAGAATCCCTTG-3’ |  |
| *lip2*-up | 5'- GCGCCACCTGGTGTGCCTG-3’ |  |
| *lip2*-down | 5'- GTCGGGGTCCAGGGTCTCC-3’ |  |
| *hsiJ2*-up | 5'- CGCCCACGCATCCGCCTAC-3’ |  |
| *hsiJ2*-down | 5'- CATCATGAAATCGCCAACCTCCG-3’ |  |
| *dotU2*-up | 5'- CGAGGACGAGAAGACCGTCCTCC-3’ |  |
| *dotU2*-down | 5'- GACGTTGAAGGCCTCGCCCG-3’ |  |
| *icmF2*-up | 5'- CCACCGTGTTTCCGCCCAAAG-3’ |  |
| *icmF2*-down | 5'- CAGATTCAGCATCAGGTAGGCCCG-3’ |  |
| *stp1*-up | 5'- CATCGGCAGTACGGTGGTGG-3’ |  |
| *stp1*-down | 5'- GCGGGCCTGCTCGATATCC-3’ |  |
| *stk1*-up | 5'- GCGCTGAAGCTGCTCAACGAG-3’ |  |
| *stk1*-down | 5'- CCTTCGGGACGCTCGCAGAG-3’ |  |
| *clpV1*-up | 5'- GTGCAGAGTTCCGAGA-3’ |  |
| *clpV1*-down | 5'- GGATGGCCTTGTGCTC-3’ |  |
| *vgrG1*-up | 5'- CTCGGCTTTTCCGATTTC-3’ |  |
| *vgrG1*-down | 5'- CCAGTCGAAGAAGTGGTC-3’ |  |
| *clpV3*-up | 5'- CTGGAAAGCCTGCGCCAT-3’ |  |
| *clpV3*-down | 5'- TGCGACCGTCCAGTTCCT-3’ |  |
| *vgrG3*-up | 5'- GGCGGTTTCAACGAGCTG-3’ |  |
| *vgrG3*-down | 5'- TCACCGTGCGCGTGTCCT-3’ |  |
| promoter of *relA* | 5'- CTCGATCCGCCGCGCGACGGCGCGTTCGAGGCGGTAC  GCGAAATGAGTTCTCTGGGTGCCCGGAGGGTAGTCTATGTA  TCCTGCAATCCGGCGACCCTGGCGCGCGATGCTGGCGAAA  TGGCCAGGCAGGGCTATCGTCTGAAGCGCGCCGGAATCCT  CGATATGTTTCCGCAGACCGCACATGTCGAGGCCATGGCTT  TGTTCGAGGCAGGCTAGGATGCCTGCGTAATCCGACCAGCC  CGTTCGAAGGCTGGCGCAGTCATCGGTGGGCTACGCGCACC  GTGGTAAAGGGTAGGCAAG-3’ | EMSA probe sequence labeled with biotin on the 5' and 3’ ends |
| promoter of H2-T6SS | 5'- CTATTGGGATTTTCGTTCGGTTTTTATGCCAAC  TAGTTGAATTGTTAAGATATTCATTGGCGCACATG  GTGTGCATTG-3’ | EMSA probe sequence labeled with biotin on the 5' and 3’ ends |
| Δ*clpV2*-up-F | 5'- GGGGTACCGAGTTCTTCGAAGAGCG-3’ | primers for *clpV2* gene deletion |
| Δ*clpV2*-up-R | 5'- GCTCTAGACCTTAATGAATCTTGCTG-3’ |  |
| Δ*clpV2*-dw-F | 5'- GCTCTAGAGGTGGGTGCGATGTTCA-3’ |  |
| Δ*clpV2*-dw-R | 5'- CCCAAGCTTCAGTTCTGCACCACGAAC-3’ |  |
| Δ*rpoS*-up-F | 5'- CGGAATTCTCGGTCAAGCTATCCAAT-3’ | primers for *rpoS* gene deletion |
| Δ*rpoS* -up-R | 5'- CGGGATCCGTCGTTATCCCTTGCATG-3’ |  |
| Δ*rpoS* -dw-F | 5'- CGGGATCCGTGAGGTGGTGATTCGC-3’ |  |
| Δ*rpoS* -dw-R | 5'- GCTCTAGACTTCAGTCCCTCGTCATC-3’ |  |
| Δ*clpV2::clpV2*- F | 5'- CCCAAGCTTATGGATGAGCATGATCAAC-3’ | primers for *clpV2* gene complementation |
| Δ*clpV2::clpV2*-R | 5'- CGGAATTCTCAGGCGAACTCGCAGAGC-3’ |  |
| P*_relA_*-up-F | 5'- CGGAATTCCGGCGCCGCGCTCGGAT-3’ | primers for *relA* promoter swapping |
| P*_relA_*-up-R | 5'- CGGGATCCCAGAACAGGTCGAGGACC-3’ |  |
| P*_groEL_*-F | 5'- CGGGATCCCTGGCGCGCCCGCGAAC-3’ |  |
| P*_groEL_*-R | 5'- CGTCTAGAAGTCGTAACTCTCCCAA-3’ |  |
| P*_relA_*-dw-F | 5'- CGTCTAGAGTACAGGTGAGAGCGCA-3’ |  |
| P*_relA_*-dw-R | 5'- CCCAAGCTTACTTCCCGGGCGACGCG-3’ |  |

**Supplementary Information of Methods**

The supplementary method information of bacterial survival assay: In this experiment, bacteria (the wild-type strain and Δ*lasR* strain) were cultured overnight. Then, we set different groups, and each group contained at least three replicates. Bacterial suspensions were prepared to be treated in different groups. Then, after standing at room temperature for different hours, we performed the 10-fold serial dilution on these bacterial suspensions. We used 200 μl diluted bacterial suspensions to perform the bacterial coating on the LB solid medium. After being cultured for 12 hours, the numbers of bacterial colonies were counted. According to the formula, log_10_ (CFU/ml) = log_10_ (5×count number×10^dilution times^), the result was obtained from the bacterial colony counting. The supplementary information of MIC assay method: Based on the result of the MIC assay with two-fold dilution, we set more kanamycin concentration gradients aiming to obtain a more accurate result. And this operation used in some MIC assays is based on the MIC assay method described in Materials And Methods of the main text. The supplementary information of the continuous repeated transfer assay method: In this experiment, 10 μg/ml (0.5 U/ml) MomL was also used. Other steps in this experiment are described in Materials And Methods of the main text.
